# Contact-induced molecular reorganisation in a model *Escherichia coli* lipid membrane

**DOI:** 10.1039/d6sm00923a

**Published:** 2026-10-21

**Authors:** Nicolo Tormena, Teuta Pilizota, Kislon Voïtchovsky

**Affiliations:** a Physics Department, Durham University South Road Durham DH1 3LE UK kislon.voitchovsky@durham.ac.uk; b School of Biological Sciences and Centre for Engineering Biology, The University of Edinburgh Alexander Crum Brown Road Edinburgh EH9 3FF UK; c Department of Physics, University of Cambridge JJ Thomson Avenue Cambridge CB3 0HE UK tp579@cam.ac.uk

## Abstract

The nanoscale composition, molecular organisation and mechanics of biological membranes underpins their function for cellular compartmentalisation, signalling, and mechanical integrity. Eukaryotic membranes can readjust their molecular content and structural organisation in response to specific interactions or changes in their environment, including through the formation of more ordered and solid-like nanodomains – lipid rafts. Recent studies have shown that bacterial membranes also possess significant lipid diversity and can form functional microdomains reminiscent of eukaryotic lipid rafts, despite lacking sterols and sphingolipids. In all cases, the role of biochemical interactions between lipids has long been recognized as determinant for the membrane molecular organisation and physical properties. In this study, we explore how non-specific physical contacts between a model lipid membrane mimicking the composition of the inner membrane of *Escherichia coli* and different substrates affect membrane molecular organisation and physical properties at the nanoscale. Using atomic force microscopy, we track the evolution of the membrane when kept a few degrees above its transition temperature. The sample is fully immersed in a solution mimicking the ionic composition of the standard *E. coli* growth media and placed in a sealed chamber to prevent changes in the interfacial hydration layer between the membrane and substrate. Aside from the expected role of the substrate on the lipid phase transition, we observe a slow substrate-dependent molecular reorganisation of the membrane following a spinodal-like behaviour. Over a period varying between minutes for rougher substrates to hours for atomically flat substrates, mechanically stiffer yet still fluid nanodomains emerge. Concomitant changes in height difference between lipid nanodomains are consistent with a substrate-biased lipid flip–flop mechanism resulting in compositional asymmetry between leaflets. The results support previous theoretical predictions and demonstrate that membrane–surface interactions may be sufficient to passively drive molecular reorganisation in bacterial membranes, with consequences for membrane function and mechanics, including in processes like osmotic pressure regulation.

## Abstract 2

Physical contact with surfaces can reorganise model *E. coli* membranes, driving lipid redistribution and the emergence of nanodomains with distinct mechanical properties.

## Graphical abstract

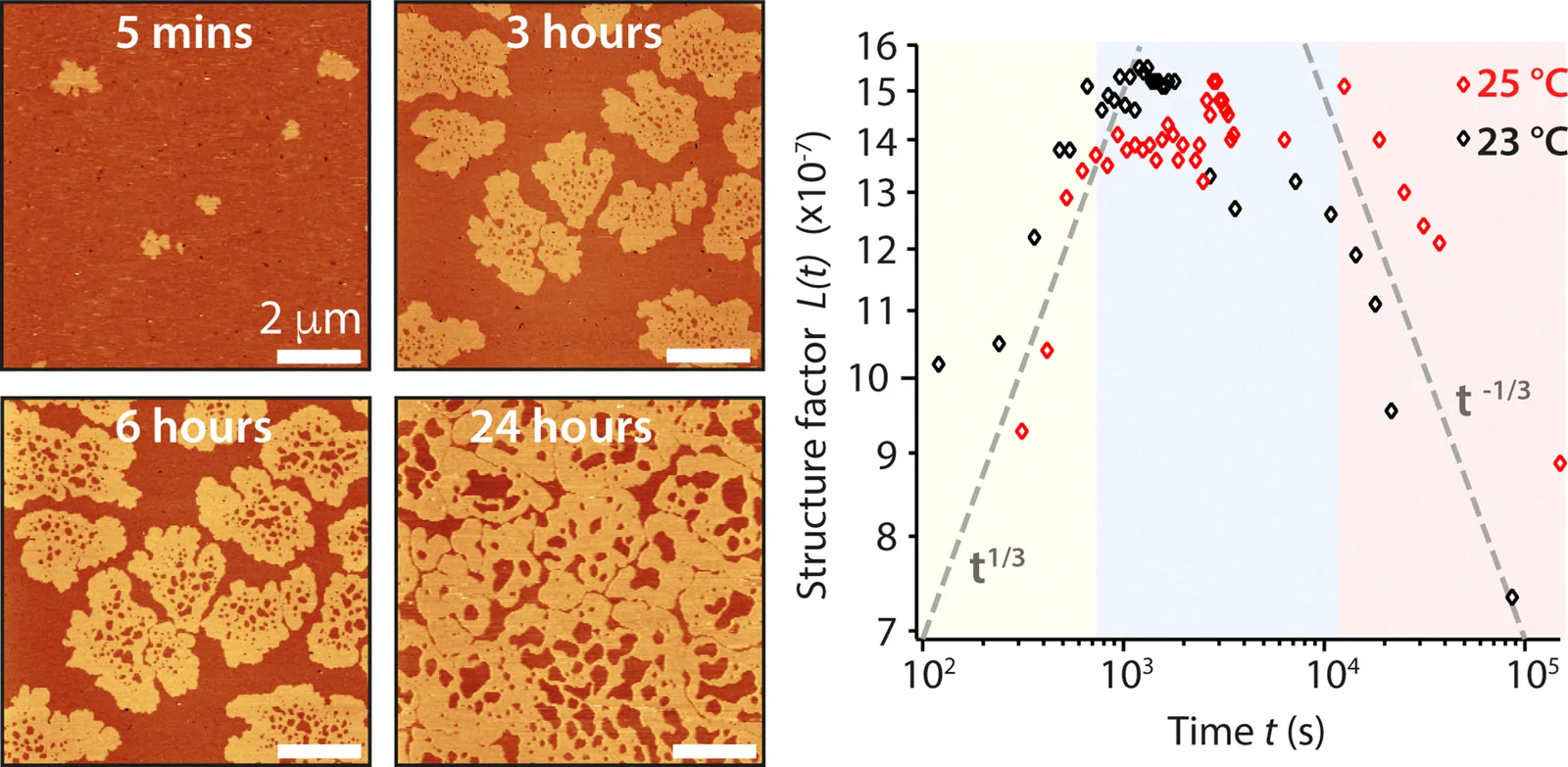

## Introduction

Biological membranes compartmentalise the cell and separate it from the external environment. They are essential for cellular homeostasis, signalling and uptake of macromolecules and ions, as well as bearing mechanical load.^1–3^ Compositionally, biological membranes are lipids arranged in a bilayer, together with other amphiphilic molecules, and contain a diverse array of embedded proteins and associated carbohydrates.^1,2^ Extensive research has focused on the relationship between the compositional complexity of biological membranes and the resulting molecular organisation and structure across scales.^4–7^ Early studies on model membrane systems composed solely of lipids revealed distinct physical phases that depend on structural properties of the lipids,^8,9^ for example from tightly packed solid-ordered (*S*_o_) to more fluid, disordered phases termed liquid disordered (*L*_d_). Subsequent studies on plasma membranes of eukaryotic cells indicated the existence of intermediate liquid-ordered (*L*_o_) phase domains, where lipids are packed tighter than *L*_d_ phase but retain a degree of lateral mobility.^10,11^ Because such *L*_o_ domains, often termed lipid rafts, are small and temporally dynamic structures, their existence has been extensively debated over the last decade.^12^ The current consensus not only recognizes their existence *in vivo*, but also their role in regulating the uptake of extracellular vesicles,^13^ mechanosensing^14^ and other signalling processes,^15,16^ including hosting and regulating crucial membrane receptors for pathogen recognition.^17,18^ Lipid rafts have a common lipidomic profile consisting of phospholipids or sphingolipids mixed with sterols such as cholesterol.^19^ Cholesterol, in particular, has been extensively studied for its role in modulating the fluidity of lipid rafts and the characteristics of the *L*_o_ phase.^20,21^ Because cholesterol is a defining feature of eukaryotic cells, the emergence of lipid rafts has often been viewed as a critical evolutionary step.

In contrast, most prokaryotic membranes lack both sphingolipids and sterols and were long thought to be structurally simple. However, several studies have shown that bacterial membranes exhibit a far more diverse lipid composition than previously assumed.^22–24^ Bacterial lipids are remarkably varied, a complexity that has often been overlooked due to the dominance of nutrient-rich culture studies in the literature.^22^ Under stress or nutrient-depleted conditions, bacteria display extensive lipid diversity and engage in homeoviscous adaptation, a process by which they modify their membrane composition to maintain fluidity across different environmental conditions.^22^ Furthermore, a growing number of studies on bacterial membranes provide strong evidence for functional, lipid raft-like, microdomains.^25–29^ In most microbes, their formation is driven by lipid–lipid interactions, particularly in the presence of cardiolipin,^26,30,31^ carotenoids,^32,33^ and hopanoids,^34–36^ which behave similarly to cholesterol and cluster within native lipid domains, also modulating the local membrane fluidity.^26,31,37^ The implication is that the *L*_o_ domains in biological membranes predates the evolution of sterols,^36^ with the exception of *Borrelia burgdoreri*.^38^

Beyond lipid–lipid interactions, the biological membrane is not an isolated free-standing layer but rather exists within a highly crowded cellular environment and is itself crowded with proteins.^6,39^ These proteins, whether embedded within the bilayer or associated with external scaffolds and filamentous networks, interact with the bilayer and play a role in modulating the membrane's functional, mechanical, and topographical properties, as well as contributing significantly to their lateral organisation.^40–44^ Even minimal physical contacts can significantly influence membrane domain behaviour^45^ or trigger local phase transitions or molecular reorganisation in the membrane.^44^

However, most of our understanding of this interplay comes from studies in eukaryotic systems, despite the bacterial intracellular environment being even more crowded, and the recent evidence that *E. coli* membranes can bear mechanical load.^3,46^ Furthermore, the bacterial cytoskeletal network,^47^ which comprises homologues of eukaryotic actin and tubulin proteins, remains in close contact with the inner membrane throughout key cellular processes such as division,^48^ proliferation,^49^ morphogenesis,^50,51^ and motility.^52,53^ Other membrane-proximal structures, such as the peptidoglycan layer,^54,55^ termed the cell wall, are also in contact with membranes. The cell wall plays a shaping role and bears osmotic pressure, making it a target for many antibiotics.

While specific interactions between the membrane and contacting structures have been the focus of multiple studies,^50,51,55^ the fundamental aspects of non-specific ‘physical’ contact on the local organisation and evolution of bacterial membranes remain poorly understood. With the main transition temperature of bacterial membranes usually close to that of their environment,^22^ small perturbations can in principle induce large changes.^44,56^ It is, for example, well-known that the stabilising effect of a substrate affects the membrane's phase behaviour and increases the main transition temperature (*T*_m_) by up to ∼10 °C.^57–60^ The transition can be decoupled between leaflets in supported lipid bilayers (SLBs), with the proximal leaflet – closest to the substrate exhibiting the highest *T*_m_.^57–59,61^ This occurs despite a 1–2 nm thick water layer present between the substrate and the proximal leaflet.^62,63^ At the molecular level, studies have shown that water- and ion-mediated interaction with a substrate can alter diffusion in the proximal leaflet^64^ and lead to molecular redistribution within leaflets.^61,65–68^ A theoretical study^69^ has suggested that interactions with the substrate can bias the exchange of lipids between leaflets – the so-called lipid flip–flop – potentially resulting in membranes with asymmetric lipid composition. However, the effect of the substrate on the flip–flop process is not yet fully understood with multiple parameters such as membrane curvature, pressure or the presence of defects all influencing experimental results.^70–74^

Using atomic force microscopy (AFM) in liquid, here we investigate the impact of different substrates on the longer-term evolution (over more than 24 h) of an all-lipid model system of the inner membrane of *E. coli*, when kept 3 °C to 5 °C above *T*_m_. The choice of temperature reflects real-life situations with bacterial membranes naturally and actively maintaining their *T*_m_ a few degrees below the environment,^22^ while amplifying the impact of the substrates on the phase behaviour of the membrane.^57–59,61^ Our results extend beyond established substrate-induced modifications of the main phase transition^57–60^ to reveal a longer-term evolution of the membrane following the initial transition. This evolution is characterised by a distinct spinodal-like molecular reorganisation into nanodomains, occurring over strongly substrate-dependent timescales. Comparison between AFM and differential scanning calorimetry (DSC) conducted on suspended vesicles with the same composition highlights the striking dependence of the phase transition kinetics on the substrates, even in the absence of any canonical *L*_o_ promoting components. No structural evolution is observed for the suspended membranes, suggesting that the changes are induced by non-specific water- and ion-mediated contacts with the different substrates. Ions such as Na^+^, K^+^, Ca^2+^ and Mg^2+^ are present in the solution and between the membrane and substrate but their concentration and hence their role is kept constant throughout the experiment. The existence of a general mechanism for the spontaneous formation of nanodomains in prokaryotic membranes, and in environments with characteristic ionic composition can have far-reaching consequences for our understanding of their organisation and function, with the present work providing novel insights into their native morphology, including the recently discovered role they play in actively bearing osmotic pressure.^3,46^

## Materials and methods

### Lipids and chemicals

The commercially available lipids, 1-palmitoyl-2-oleoyl-*sn*-glycero-3-phosphoethanolamine (POPE) and 1-palmitoyl-2-oleoyl-*sn*-glycero-3-phospho-(1′-rac-glycerol) (POPG), were purchased and dissolved in chloroform from Avanti Polar Lipids (Alabaster, USA).

Salts (all > 99% purity) were purchased from Sigma-Aldrich (Gillingham, UK) and dissolved in ultrapure water (Merck-Millipore, Watford, UK) to prepare MOPS buffer-based solution with specific ion concentrations mimicking standard *E. coli* growth media^75,76^ as follows: 50 mM NaCl, 9.5 mM NH_4_Cl, 0.5 mM MgCl_2_, 0.3 mM K_2_SO_4_ and 1 µM CaCl_2_–2H_2_O. The pH was adjusted to 6.5 prior to mixing with lipids.

### Large multilamellar vesicles preparation

10 mg of lipids dissolved in chloroform were mixed in a 4 mL glass vial following the correct molar ratios. To allow chloroform evaporation, the lipids were then pre-dried under a gentle nitrogen flow and finally placed overnight in a vacuum chamber. Lipids were rehydrated with 1 mL of MOPS buffer-based solution obtaining a final concentration of 10 mg mL^−1^ and large multilamellar vesicles were obtained through freeze-thawing. Briefly, the lipid solution was heated while sonicating in a bath sonicator and subsequently, the lipids were frozen by leaving the glass vial for 15 min in a freezer. Six consecutive cycles of heating–freezing were repeated to ensure vesicles formation, as indicated by the lower turbidity of the final solution.

### Differential scanning calorimetry (DSC) measurements

To observe the effects of the scan rate on the thermodynamic properties of lipid vesicles, DSC measurements were performed on a DSC 2500 (TA Instruments, Delaware, USA). 10 µL of LMVs solution at 10 mg mL^−1^ were loaded into the calorimeter and the sample was equilibrated before the measurement for 5 min at the starting temperature. DSC runs on vesicles were performed with varying cooling rate (from 0.03 °C s^−1^ up to 0.67 °C min^−1^) within a temperature range of 60 °C to −10 °C. DSC runs were repeated 3 times per sample to ensure reproducibility.

To test compositional rearrangement in the vesicle's membrane, a series of DSC cycles were performed with the same DSC 2500 instrument. After loading the LMVs in the same volume and concentration as previously described, the vesicles were equilibrated at 45 °C for 5 min. The sample was then cooled down to 16 °C at a rate of 0.08 °C s^−1^ and held at that temperature for set amounts of time. At the end of this isothermal phase, the sample was heated back up to 45 °C with a rate of 0.08 °C s^−1^ and equilibrated at that temperature before the start of the next cycle.

### Small unilamellar vesicles preparation

Similarly to LMVs preparation, lipids were mixed and dried in a 4 mL glass vial before being rehydrated in the MOPS buffer-based solution. The lipid solution was gently bath sonicated for 15 min at 45 °C, until the solution looked opaque and milky, indicating the dissolving of lipids in the solvent. The solution was extruded 31 times using a Mini-Extruder kit (Avanti Polar Lipids through Sigma-Aldrich) with 2 Whatman 100 nm filter (GE Healthcare Life Sciences, Little Chalfont, UK) to form small unilamellar vesicles (SUVs). Appropriate final SUVs concentration was prepared depending on the surface used for the supported lipid bilayer preparation.

### PDMS surface preparation

Sylgard 184 silicon elastomer kit was purchased at Dow Corning Corporation (Midland, USA).

Silicon elastomer and curing agent were gently mixed in a 10 : 1 weight ratio in a glass container. Subsequently, the mixture was degassed in a vacuum chamber for at least 1 h to remove any air bubble in the solution. The PDMS solution was then deposited on top of a cleaved Muscovite mica disk (SPI Supplies, West Chester, USA) and placed inside a WS-650 Mz-23NPPB spin-coater (Laurell Technologies Corporation, Landsalle, USA) to allow a homogenous distribution of the PDMS over the surface. Spin-coating was performed for 10 min at 1000 RPM at room temperature. Finally, the PDMS-coated disk was placed in an oven at 50 °C overnight to ensure the curing of the PDMS layer. The PDMS layer was subsequently oxidised with plasma (1 mbar, 300 W VacuLAB, Tantec, Redditch, UK) for 30 s immediately before lipid deposition in order to ensure suitable bilayer formation.^77^

### SLB and monolayer preparation on different surfaces

The SUVs solution was diluted to an appropriate concentration to ensure full bilayer or monolayer formation depending on the surface used. To form SLB on a mica surface, 100 µL of 0.2 mg mL^−1^ SUV solution were deposited on a freshly cleaved Muscovite disk (SPI Supplies, West Chester, USA) placed on the AFM stage and left to incubate for 30 min at 50 °C covered with a Petri dish. To form the SLBs on PDMS, 100 µL of 1 mg mL^−1^ SUV solution were deposited on the freshly oxidised PDMS surface for 30 min at 50 °C. Finally, to form a lipid monolayer on top of highly ordered pyrolytic graphite, 100 uL of 0.4 mg mL^−1^ SUV solution were deposited on freshly cleaved HOPG surface (SPI Supplies) for 30 min at 50 °C. All the samples were subsequently gently rinsed with the solvent to remove any unfused vesicle, and the temperature was lowered to 40 °C for 15 min before starting the experiment.

### AFM measurements

Imaging was conducted using a commercial Cypher ES AFM (Oxford Instruments, Santa Barbara, USA), equipped with temperature control (heating/cooling sample mount with feedback). SNL-10 cantilevers (Bruker Scientific instruments, Billerica, USA) with nominal spring constant of 0.35 N m^−1^ were used. The tip has a pyramidal shape with a nominal radius ≤12 nm at its apex. The AFM imaging was performed in amplitude mode, where the tip was acoustically oscillated at a frequency close to its resonance while fully immersed in the liquid. By adjusting cantilever oscillations, the imaging conditions were kept as soft as possible ensuring neither tip contamination nor damaging of the sample. Force spectroscopy curves and map were acquired by working in contact mode with a SNL-10 cantilever. Force maps were created from 1024 force curves (32 × 32) over a 6.25 µm^2^ area. Through the acquired force curved, membrane's mechanical features such as *F*_R_ and *Y*_M_ were calculated based on commonly used AFM force spectroscopy approaches with thin biological systems.^78^ The Young's modulus, *Y*_M_, has been calculated through a corrected version of the well-established Hertzian indentation model for a semi-infinite medium^79^ that takes into account the finite thickness of the bilayer and the presence of a hard substrate underneath it.^78^ To limit the surface contribution the indentation depth was kept below 20% of the bilayer's thickness. We note that commonly used assumptions for the model (*e.g.* incompressible membrane) are not necessarily true for biological systems.^80^ To still allow for quantitative comparison, we used the same cantilever/tip for all the maps. The cantilever was calibrated before and after the experiment. The inverse optical lever sensitivity and the spring constant were determined by recording a force–distance curve on a stiff mica surface and through the cantilever's thermal spectrum, respectively.

### Data analysis

DSC results were analysed with the TRIOS Software, provided with the instrument. The software was used to normalize the raw heat flow data to the sample mass (this “normalized” heat flow is referred to just as “heat flow” in this work), correct thermogram baselines, and extract the corresponding thermodynamic parameters. Here, *T*_m_ was identified at the highest point of each calorimetric peak. The calorimetric enthalpy change was calculated by integrating the area under the thermal peak, and the on-set temperature was identified as the first portion of the curve deviating from the DSC baseline. When comparing the shape of the cooling and heating DSC profiles, a baseline was subtracted (linear fit) and the curves then normalized with respect to their respective maximum.

AFM images were flattened to remove background slope and noise, and analysed using the Gwyddion software,^81^ an open-source modular program for scanning probe microscopy data visualization and analysis. The structure factor, *S*_k_ and subsequently *L*(*t*) were calculated from AFM images using a custom-written script in Igor Pro (Wavemetrics, Lake Oswego, USA). Prior to analysis, AFM images were flattened by subtracting a fitted 1st order polynomial background from each scan line, followed by a 2D Fast Fourier transform used to derive *S*_k_ through radial averaging in *k* space. To ensure meaningful interpretation, the *k* values corresponding to features larger than ½ of the AFM images were progressively removed to avoid edge effects. To determine the scaling behaviour of *L*(*t*) *versus t*, linear fits were performed on log–log plots, where the power–law relationships appear as straight lines. Fitting regions were selected based on both visual inspection done to identify apparent power–law regimes, and a more systematic approach as follows. For the initial regime, a linear regression with a fixed slope of 1/3 was fitted over progressively larger intervals, and the fit was extended until the *R*^2^ value dropped below an acceptable threshold (*R*^2^ < 0.98). An analogous approach was applied to the final portion of the curve, using a linear regression with a slope of 1/3.

AFM force spectroscopy data were fitted using a custom script written in Igor Pro, allowing for the automated identification of the *F*_R_ and *Y*_M_.^82^ Analysis of height variation between domains was also carried out using bespoke routines in Igor Pro, focusing exclusively on the boundary region between domains (more details are available in the supplementary information (SI)). Graphs were generated using Igor Pro Software and in Python.

## Results

With the stated aim of investigating the general mechanisms underpinning contact-induced changes in the molecular behaviour and evolution of prokaryotic membranes, some simplifications are needed. The complexity of native membranes makes it difficult to identify passive mechanisms affecting the whole membranes from specific biochemical interactions or active processes driven by proteins. We therefore selected a lipid-only model system for the inner membrane of *E. coli*. Multiple model systems have previously been proposed (see^82^ and references therein), comprising a mixture of zwitterionic phosphatidylethanolamine (PE) lipid headgroups, anionic phosphatidylglycerol (PG) and cardiolipin. Our recently developed model system of 75% POPE, 20% POPG and 5% cardiolipin, mimics the lipidomic composition, *T*_m_ and mechanical properties of the inner membrane of *E. coli*,^82^ making it particularly relevant for the present study. However, since cardiolipin has a well-established propensity to promote the formation of lipid nanodomains,^25,30,31^ using it here would introduce an additional mechanism for lateral segregation. We therefore deliberately excluded it and opted for a binary POPE/POPG system enabling us to test whether contact-induced reorganisation can occur in the absence of this known domain-promoting lipid. Furthermore, we selected MOPS-based buffer with an ionic composition typical of *E. coli* cultures.^75,76^ This lipid composition and environment still replicates the phase transition of the inner membrane of *E. coli*^82^ and offers a direct point of comparison with previous work^58^ while allowing us to study general, contact-induced effects on lipid organisation.

### Verifying the impact of surface interactions on the kinetics and equilibrium of the main phase transition

Before examining the kinetics of the phase transition, we first verify the anticipated substrate-induced decoupling of the leaflet during the transition.^57,58,61,65,83^ The effect is usually understood as resulting from a competition between lipid–substrate and lipid–lipid interactions. Here we compare the phase transition (upon cooling) of suspended vesicles in solution using DSC, and mica-SLBs with AFM, revealing an overall good agreement with previous studies^58^ (see SI Fig. S1 for details). The inferred equilibrium transition temperatures are *T*_m_ = 20.0 °C ± 0.1 °C for suspended vesicles, 27.2 ± 0.6 °C for the proximal leaflet of the SLB and <15 °C for the decoupled distal leaflet. For the SLBs, the equilibrium values were obtained by allowing 15 min thermal equilibrating after every cooling step of 2 °C (average cooling rate is 10^−3^ °C min^−1^, see SI Fig. S2 for details). Any small differences with previously reported *T*_m_ values^58^ can be explained by the different ionic environment used here: the MOPS buffer relevant for *E. coli* cultures (see Materials and methods).

Repeating the experiment at different cooling rates offers a quantitative picture of the transition kinetics for both unsupported and supported membrane (Fig. 1). For the SLBs we focus exclusively on the proximal leaflet given its enhanced sensitivity to the contacting substrate.

**Fig. 1:**
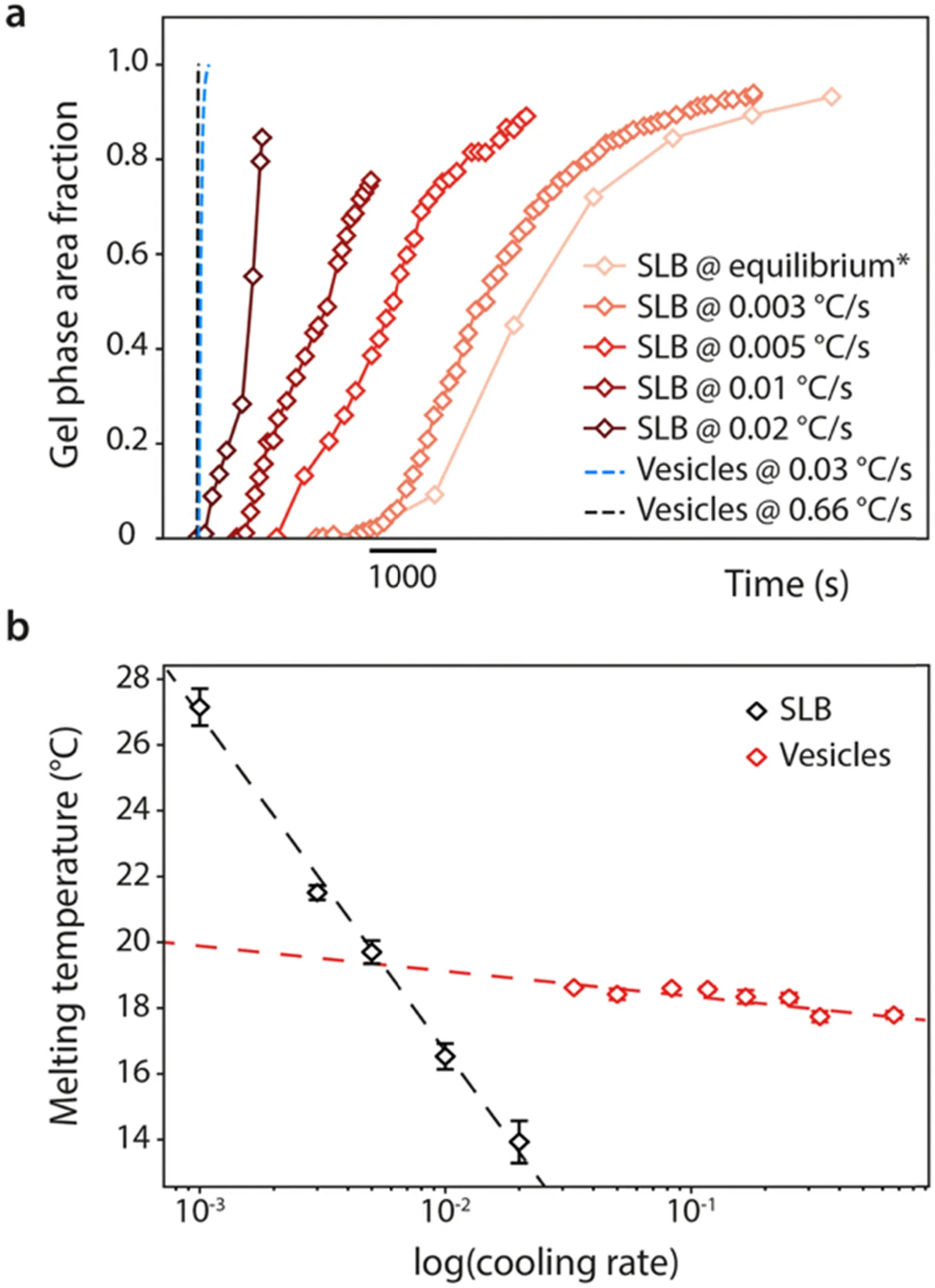
Comparative phase transition kinetics for unsupported and supported bilayers upon cooling. (a) The progression of the transition *vs.* time is shown for different cooling rates, both for unsupported large vesicles (dashed lines) and the proximal leaflet of SLBs (diamond marked lines). The SLB curves have been horizontally offset for clarity, with *T*_m_ identified as the temperature where 50% of the membrane (or leaflet) is in the gel phase. (b) The derived *T*_m_ values vary exponentially with the probed cooling rates (see SI Fig. S2 for more details). The error bars represent the standard deviation.

The exponential dependence of *T*_m_ on the cooling rate, clearest for the SLB's proximal leaflet (Fig. 1b), highlights the dramatic impact of the substrate on the dynamics of molecular changes in the membrane. This result is all the more remarkable considering the relatively weak interaction between lipid headgroups and the solid substrate underneath, which are separated by several layers of water with an overall water thickness ranging from 1 to 2 nm.^62,63^ We note that the ions trapped between the membrane and the substrate are known to influence the membrane–substrate interactions,^58,68^ especially divalent ions.^84^ However, the ionic concentration and hence the impact of these ions is kept constant throughout the experiment. The exponential dependence should breakdown at slower cooling rates, with *T*_m_ eventually reaching a rate-independent maximum value. Nonetheless, Fig. 1 highlights the fact that molecular processes in the proximal leaflet of SLBs can take significant time (>hours) to reach equilibrium, opening the possibility of spontaneous processes such as molecular exchanges with the solution or flip–flop,^70,72^ changing the phase behaviour of the system.

### The substrate-induced molecular reorganisation takes place over hours, affecting structure and mechanics

To explore the possible longer-term evolution of the mica-supported SLB, we keep the membrane at temperature *T*_c_ several degrees higher than *T*_m_ in solution, but lower than the expected *T*_m_ for the proximal leaflet of the SLB at equilibrium. Practically, the system is first equilibrated for >30 min at 40 °C (far above its *T*_m_), and then rapidly (1 °C s^−1^) quenched to *T*_c_ where it is kept for 24 h. The SLB remains continuously immersed in the MOPS buffer solution, in a sealed and humidity-saturated chamber. We conducted two separate experiments, either with *T*_c_ = 25.0 ± 0.1 °C or with *T*_c_ = 23.0 ± 0.1 °C. As a control, the experiment is also conducted on a pure POPE bilayer kept at *T*_c_ = 30.0 ± 0.1 °C (*T*_m_ = 25 °C in solution), revealing a simple and complete phase transition of the whole proximal leaflet (SI Fig. S3), albeit with the relatively slow kinetics expected from lipid–substrate interactions. A significantly more complex behaviour is visible for our model binary membrane of *E. coli* in MOPS buffer solution (Fig. 2a). Early stages of the transition follow the expected nucleation and growth behaviour, with initially small, randomly distributed *S*_o_ proximal domains that grow over time. However, between 1 and 3 h we observe a slowdown in the growth of the *S*_o_ domains, coupled with the formation of new thinner domains in the middle of the *S*_o_ domains. The reduced thickness implies that the proximal leaflet of these new domains is in *L*_d_ phase. Thus, the whole membrane is evolving from partially *S*_o_ to fully *L*_d_ in these regions (arrows in Fig. 2a). The observed reorganisation of the membrane not only stops and partially reverses the *L*_d_ → *S*_o_ transition, but also transforms the morphology of the domains over 24 h, in a manner reminiscent of a spinodal decomposition.^85^ Control experiments with the SLB always maintained fluid (∼4 °C above *T*_m_ of the proximal leaflet) did not show any evolution over >3 h (SI Fig. S4).

**Fig. 2:**
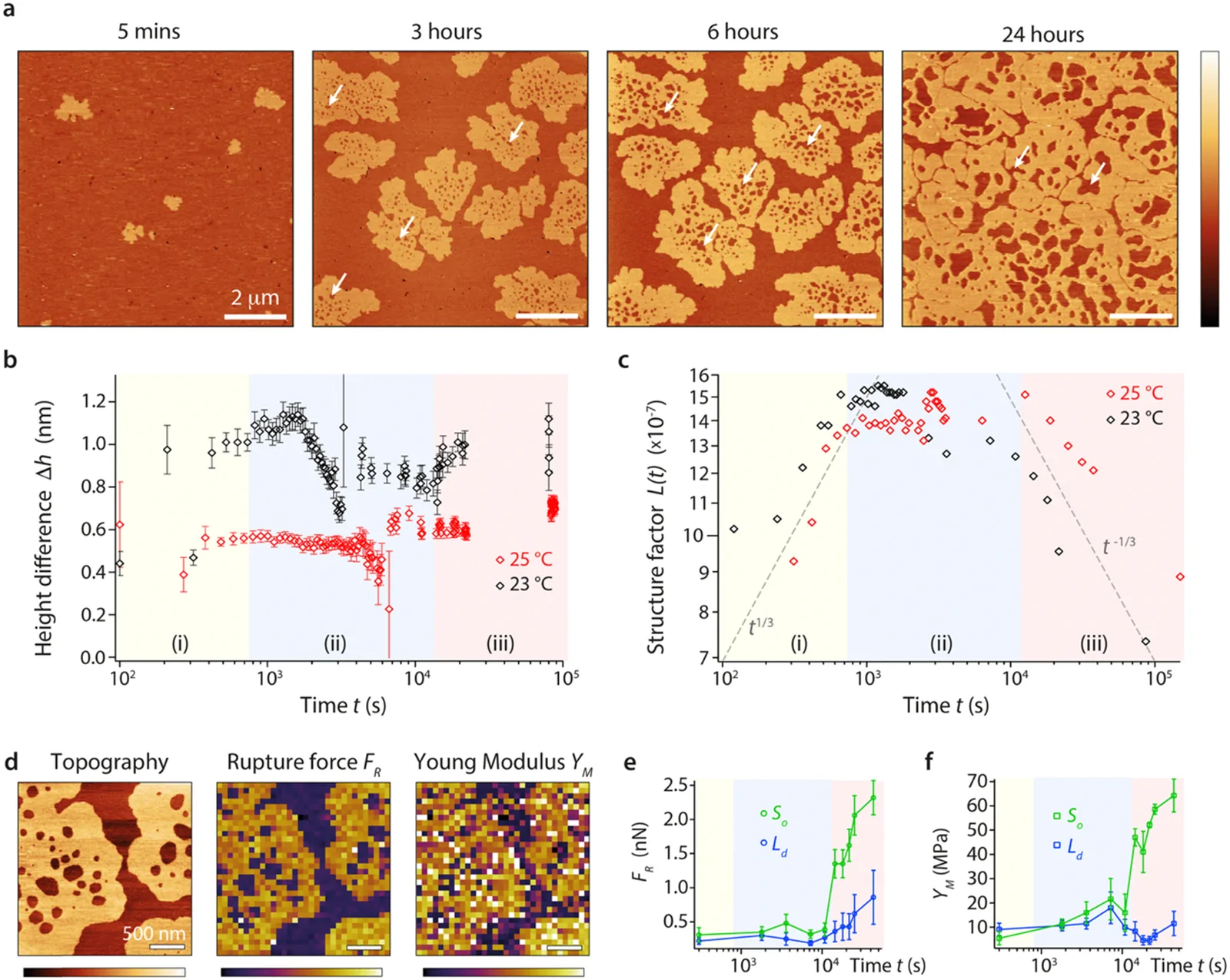
Contact-induced reorganisation of the membrane. (a) Consecutive AFM topography images of a same SLB region evolving at 25 °C reveal a molecular reorganisation of the membrane, with fluid regions (arrows) progressively appearing in the otherwise *S*_o_ proximal leaflet. (b) Height differences between higher (proximal *S*_o_) and lower (fully *L*_d_) domains (see SI Fig. S5 for details of the analysis) help us loosely define evolution regimes (shaded coloured background) with regime (ii) showing significant variations in height between domains. (c) The regimes are more clearly differentiated in the temporal evolution of the membrane's structure factor *L*(*t*) over 24 h. The log–log representation of *L*(*t*) *vs. t* shows initial growth of the *S*_o_ domains (dashed grey line representing *t*^1/3^, regime (i)), the growth stops and the onset of membrane reorganisation (regime (ii)) and the spinodal-like later evolution (dashed grey line representing *t*^−1/3^, regime (iii)). The implications of the molecular reorganisation for the membrane mechanical properties are quantified using nanomechanical mapping (d), here illustrating a measurement taken at 6 h. The corresponding maps of the extracted Young's modulus and rupture force are also shown. The same measurement repeated at set time intervals reveals a differential evolution of the membrane rupture force *F*_R_ (e) and stiffness *Y*_M_ (f) between the proximal *S*_o_ and fully *L*_d_ domains (see SI Fig. S6 for the plots of the *S*_o_/*L*_d_ ratios). The same evolution regimes are shaded. In all cases, the error bars represent one standard deviation.

To gain more quantitative insights on this evolution, we track the changes in domain thickness (Fig. 2b) and shape (Fig. 2c) over the 24 h experiment. The former are obtained by quantifying the height difference, *h*, between the highest domains (proximal leaflet *S*_o_) and the lowest domains (all *L*_d_, see SI Fig. S5 for details of the analysis procedure). Three *h* regimes can be broadly distinguished: (i) slow and monotonic increase as the proximal *S*_o_ domains nucleate and grow, (ii) subsequent monotonic decrease, followed by abrupt and irregular step-like variations and finally (iii) constant value, larger than the maximum reached in regime (i). Regime (i) reflects a stable molecular arrangement at the boundary of *S*_o_ domains that steadily grow over the first ∼1 h of the process. Regime (ii) coincides with the apparition of *L*_d_ domains inside the previously purely *S*_o_ domains, indicating a molecular reorganisation of the leaflets. The fact that *h* changes more abruptly suggests an evolving molecular composition of the domains, at least within each leaflet. Regime (iii) is consistent with a relatively stable reorganised membrane, but with domains exhibiting a different molecular composition (different *h*) than in regime (i).

Changes in the shape of the domains can be addressed through a general and system-agnostic procedure examining the scaling of the domains over time. The underlying hypothesis is the existence of a length scale *L*(*t*) ∝ *t*_*α*_ with *t* the time and *α* characteristic of the domain growth process at play.^86,87^ The definition of *L*(*t*) is not unique, but it is generally related to the structure factor *S*_k_ of the system, defined as an average over *k* values *S*_*k*_(*t*) = 〈*ϕ*(*k*, *t*)*ϕ*(−*k*, *t*)〉 with *ϕ*(*k*, *t*) the Fourier transform of the AFM image.^86,88^ We then calculate *L*(*t*) as follows:^88^1
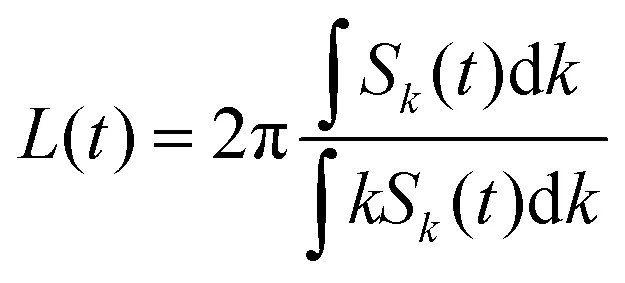


When tracking the evolution of the system supported by an atomically flat mica surface (Fig. 2a), plotting *L*(*t*) *vs. t* in a log–log representation retrieves our three regimes (Fig. 2c), providing important clues about the evolution process at hand. In regime (i), a classical diffusion-limited growth takes place, characterized by *α* = 1/3.^89^ This is followed by a plateau (regime (ii)) that coincides with the slowdown of the domain's growth and the onset of formation of the secondary domains. Finally, the third regime appears after ∼3 h with a decay in the *S*_o_ phase in favour of the *L*_d_ phase and is characterized by *α* = −1/3, a value previously observed in spinodal processes.^88^ Spinodal processes are spontaneous^87^ and do not require overcoming any energy barrier, thus the fact that it occurs in the later stages of the experiment points to particularly slow kinetics with the late formation of suitable conditions. This is consistent with a slow, substrate-induced molecular reorganisation of the bilayer, as illustrated by the evolution of *h*. We propose that it is induced by substrate-mediated inter-leaflet flip–flop of the lipids,^72–74,90,91^ resulting in domains with different lipid composition: depleted or enriched in one of the two lipid species, which were initially distributed evenly across the membrane. While the lateral and rotational diffusion occur rapidly in synthetic and native biological membranes,^92,93^ the flip–flop process is considerably slower without the involvement of enzymes,^94^ because of the high energetic cost associated with the hydrophilic headgroup passing through the hydrophobic interior of the bilayer. Consequently, passive flip–flop can be significantly affected by environmental parameters such as temperature,^95^ the presence of defects in planar bilayers,^96^ and of a solid substrate.^70,73,74^ Here, the results are consistent with the substrate influencing and biasing spontaneous lipid flip–flop, which could progressively generate compositional differences between the two leaflets.^72,95,97^ Consistently, repeating the experiment at different quenching temperatures uniformly changes the kinetics of the process through all three regimes, with slower kinetics the further the system is from *T*_m_ as seen in Fig. 2b and c.

A similar effect has previously been reported on phosphocholine membranes where an asymmetry in phase behaviour between leaflets was achieved through a careful choice of lipids.^98^ The present results suggest that the effect may be more common in mixed membranes comprising lipids with different properties and interactions with a contacting solid.

To further test our hypothesis of substrate-mediated molecular reorganisation of the membrane, we conducted nanomechanical measurement over the different domains at set times using AFM (Fig. 2d, see Materials and methods for details). A redistribution of the two lipid species is bound to affect the local membrane mechanics.^99^ Nanomechanical mapping allows sufficient spatial resolution to identify different domains unambiguously, using force spectroscopy measurements to systematically extract the membrane's Young modulus^78^ (*Y*_M_) and rupture force^99^ (*F*_R_) under pressure from the AFM tip. As expected, *S*_o_ regions appear stiffer and harder to break compared to the *L*_d_ phase.^99,100^ However, the difference is exacerbated during regime (iii) for both the average *F*_R_ (Fig. 2e) and *Y*_M_ values (Fig. 2f). Because the geometry of the AFM tip may evolve over the course of the nanomechanical measurements, we also calculated the ratio between the average values derived over the apparent *L*_d_ and *S*_o_ domains (SI Fig. S6), confirming a significant stiffening of the *S*_o_ phase compared to the *L*_d_ regions after ∼3 h, at the onset of the spinodal process.

### Substrates control the membrane evolution kinetics

The flip–flop rate is known to depend on membrane fluidity,^101^ and the presence of defects,^96^ all of which can be changed by swapping the atomically flat and regular mica for different substrates.^64^ We do so by adding a polymeric cushion of oxidized PDMS (Fig. 3), which creates a rough and uneven hydrophilic substrate that mimics the biomechanical properties of a peptidoglycan structure naturally present between the inner and outer membranes of *E. coli* (*Y*_M_ ∼ 3–50 MPa^102,103^). The PDMS also features some solution-filled gaps where the membrane is locally suspended. These more realistic features should render PDMS SLBs more prone to defects,^104^ which could facilitate flip–flop. The AFM results of the membrane evolution when supported on PDMS show a similar evolution as observed on mica, but the first *S*_o_ domains characteristic of phase (i) form too rapidly for imaging (Fig. 3a). The topographical features consistent with the spinodal process can already be observed after ∼30 min, with the formation of the *L*_d_ domains inside the *S*_o_ phase (Fig. 3a, see also SI Fig. S7 for more details). This is consistent with the hypothesis of spontaneous lipid flip–flop as a driving mechanism for the molecular reorganisation of the membrane. Unfortunately, the reduced SLB's stability on PDMS made it challenging to stabilise the imaging over large areas, especially at the onset of the evolution. Domains are already present in the first stable AFM images. The deduced *L*(*t*) suggests the system experiences significantly faster reorganisation dynamics and has already completed its evolution over the timescale of the experiment (*α* ≈ 0, see SI Fig. S8).

**Fig. 3:**
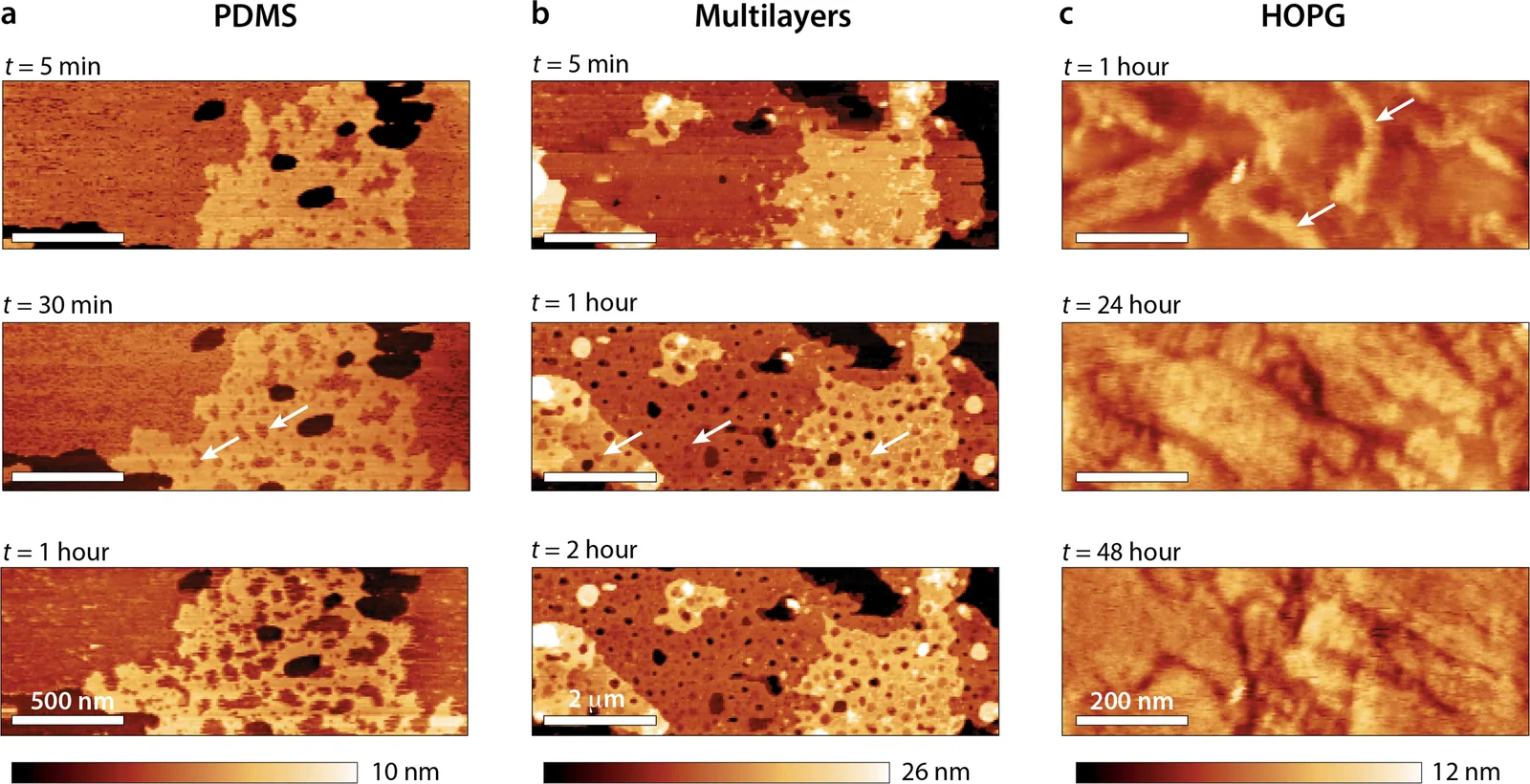
Membrane evolution on different substrates. (a) When replacing the mica substrate by a rougher polymeric surface of partially oxidised PDMS, *S*_o_ domains begin to form within less than 30 min (arrows), much faster than on mica. The molecular reorganisation is the fastest for stacked bilayers (b) with the process almost complete within less than 1 h. (c) A control experiment on HOPG shows that for a lipid monolayer made from the lipid mixture no new nanodomains appear over a 48 h period, with the monolayer progressively becoming entirely *S*_o_ from substrate step edges. Full AFM images and corresponding height profiles for each system are provided in SI Fig. S7 and S8.

We next tested the evolution of bilayer-on-bilayer structures created on the PDMS substrate (Fig. 3b). Bilayer–bilayer contacts occur naturally in *E. coli*, for example as outer membrane vesicles excretion and adsorption.^105^ More generally, the lipid mobility in the distal leaflet of the bilayer directly adsorbed to the PDMS creates a more dynamical support for the second bilayer. Together with the PDMS underneath, this strategy captures better the dynamical nature of membrane supports and has been shown to allow unimpaired function of supported proteins.^106^ Here, this also provides a direct point of comparison with our DSC measurements, which feature predominantly multilamellar vesicles (MLVs). The bilayer-on-bilayer structures exhibit the fastest evolution observed in this study, with domains appearing within minutes of quenching the temperature below *T*_m_. The evolution was too fast to conduct any meaningful *L*(*t*) analysis. These results are consistent with the idea that the mobile substrate further enhances the kinetics of the molecular rearrangement.

Finally, we conducted a control experiment with a lipid monolayer on atomically flat highly oriented pyrolytic graphite (HOPG) (Fig. 3c). We already demonstrated that the second and third regimes only occur when at least two different lipids are present on the substrate (SI Fig. S3). Here, the two lipid species are present in the monolayer, but flip–flop is not possible given the absence of a second leaflet. Additionally, the lateral diffusion of the lipids in the monolayer is significantly slower due to the enhanced interaction between the hydrophobic tails and the surface of the HOPG.^107,108^ Consistently, the monolayers on HOPG undergo a *L*_d_ → *S*_o_ phase transition of the first regime, with no appearance of heterogeneous nanodomains or spinodal process afterwards. The phase transition proceeds from the HOPG step-edges, which act as stabilisation points,^109^ and subsequently proceeds to cover the whole surface, albeit on a slower kinetics (48 h) due to the reduced lipid mobility compared to mica.^107^

### Contacts between suspended bilayers may also induce molecular reorganisation

The rapid timescale of the bilayer-on-bilayer molecular reorganisation (Fig. 3b) should, in principle, be visible in the DSC measurement, where a large proportion of MLVs are present. The contact between bilayers in MLVs can, in principle, further enhance the flip–flop induced rearrangement by allowing out of plane fluctuations and packing defects.^70^ Practically, detecting this rearrangement by DSC relies on an associated change in membrane internal free energy, affecting the shape of the DSC profile around the transition peak. Here, we explore this possibility by comparing the overall shape of the DSC cooling and heating phase transition profiles (Fig. 4, see also SI Fig. S9 for more details). The comparison reveals a larger difference in shape at the slower scanning rates of 2 °C min^−1^ and 5 °C min^−1^ (Fig. 4a), which almost disappears at 10 °C min^−1^ (Fig. 4b). This is consistent with a fast (∼1 min timescale) molecular reorganisation of the membrane that would eventually lead to identical heating and cooling profiles for even higher scanning rates. Allowing the system to equilibrate for up to 1 h at ∼2 °C below its *T*_m_ has no impact on the profile differences when subsequently re-scanned at 5 °C min^−1^ (SI Fig. S9), suggesting no slow membrane evolution on the longer term. Overall, the DSC results are consistent with the AFM results, but with 10 °C min^−1^ close to the instrumental limit in terms of sensitivity and scan rate, other possible interpretations cannot be fully excluded at this stage. We note that a direct quantitative comparison between the AFM and DSC measurements is limited by the different membrane geometries and environments. AFM probes individual planar bilayers interacting with a solid substrate, whereas DSC provides an ensemble measurement of suspended vesicles, including multilamellar structures where membrane–membrane contacts occur. The DSC measurements should therefore not be regarded as an independent validation of the molecular mechanism inferred from AFM, but rather as a complementary test of whether contact-associated membrane reorganisation can produce detectable thermodynamic changes on comparable timescales.

**Fig. 4:**
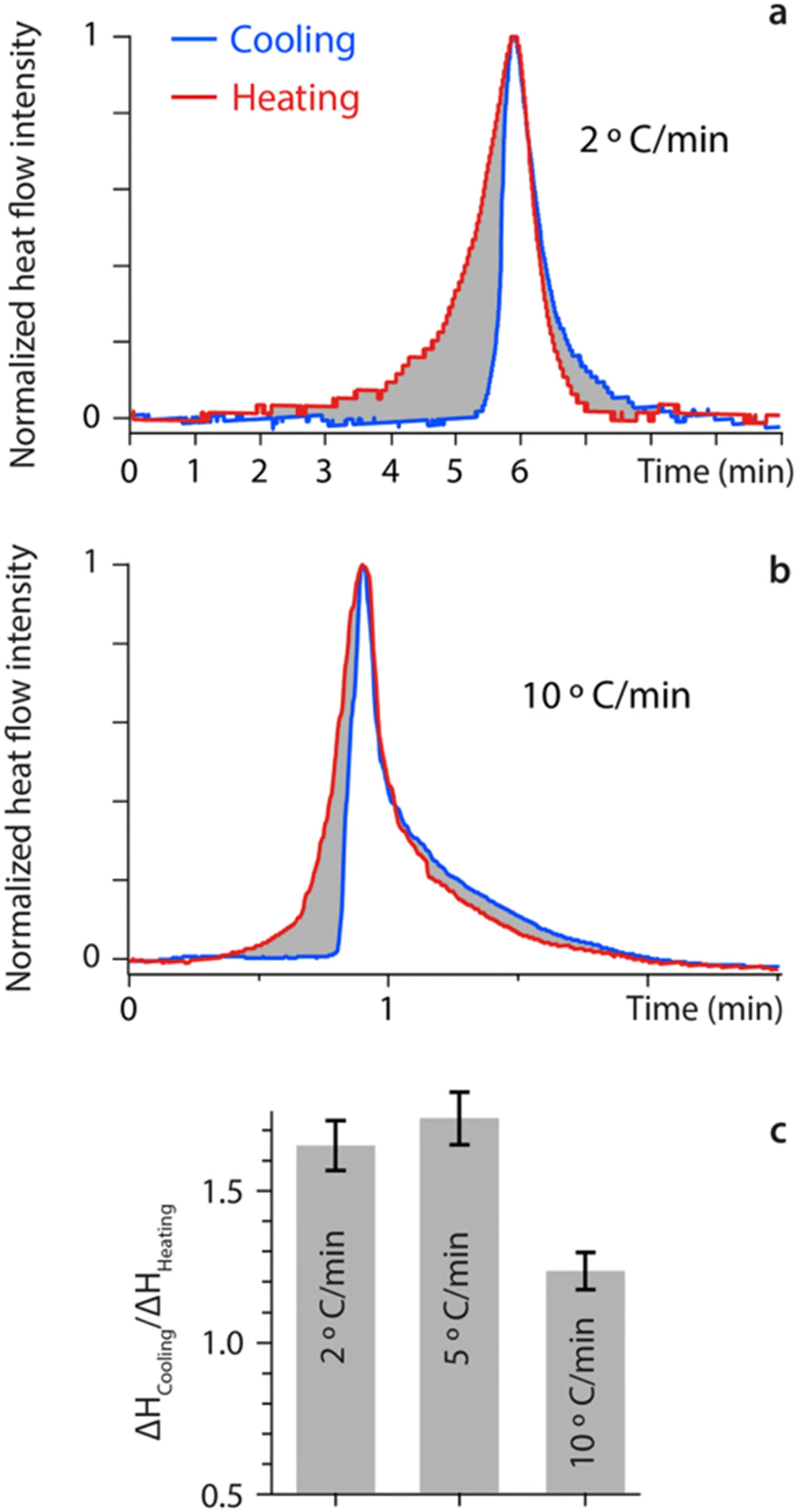
Comparison of the normalized cooling and heating phase transition profiles obtained by DSC at different rates. (a) The data acquired at 2 °C min^−1^ rate show significant differences between the normalised heating and cooling profiles (shaded in grey). At a rate of 10 °C min^−1^ (b), these differences are smaller, suggesting that the measurement time is on par with any possible membrane rearrangement. This can be better quantified by comparing the enthalpy ratio between the normalized heating and cooling transitions at different DSC scanning rates (c). The ratio is significantly smaller for 10 °C min^−1^ compared to 2 °C min^−1^ and 5 °C min^−1^, suggesting a different molecular reorganisation regime (see also SI Fig. S9 for more details on the measurement).

## Discussion

Most natural biomembranes are constantly in contact with their surroundings, from the cell wall or the cytoskeleton to compartments within the cell. Previous work has established the impact of such ‘physical’ contacts on the phase behaviour of our model *E. coli* inner membrane,^58^ showing a decoupled main transition when supported by mica in solution. The extent of this decoupling was shown to depend on the salt composition of the surrounding solution and the thermal history of the sample, suggesting the existence of multiple local minima in the energy landscape of the system. In this picture, achieving true thermodynamic equilibrium may not always be possible, or may require significantly longer periods of time. Here, we comparatively examine the kinetics of the phase transition for supported and suspended membranes as a function of the cooling rate. The results confirm the dramatic impact of the substrate on the evolution kinetics of the system, with the measured transition temperature of the proximal leaflet depending exponentially on the cooling rate and an inferred equilibrium transition >100 slower compared to the suspended membranes.

When the SLB is kept at a temperature close to, but above its transition temperature in solution, the impact of the substrate is amplified, allowing the proximal leaflet to turn into gel phase while the decoupled distal leaflet remains fluid. This state of the membrane may reflect the natural situation in the inner membrane of *E. coli* when contacting the peptidoglycan wall at room temperature.^22,54,55^ Tracking the evolution of the SLB kept at that temperature over >24 h shows that the slow phase kinetics allows another competing molecular-reorganisation process to dominate at longer timescales, with the observed behaviour being consistent with spontaneous, substrate-biased lipid flip–flop. This reorganisation is substrate-dependent and creates nanodomains with different thickness and mechanical properties than the initial membrane (Fig. 2 and 3). The existence of distinct nanodomains with different biophysical properties has previously been reported for bacterial membranes.^25–29^ In most microbes, their formation is driven by lipid–lipid interactions, particularly in the presence cardiolipin,^26,30,31^ carotenoids,^32,33^ and hopanoids.^34–36^ Here, we instead propose that the formation of the nanodomains at equilibrium (>24 h) is consistent with a compositional asymmetry generated by substrate-biased flip–flop. Ions trapped in the gap between the membrane and the substrate likely also play a role in modulating the membrane–substrate interactions,^58,68^ but with the ionic concentration kept constant throughout the experiments, these ions can be seen as part of a modified substrate. Replacing the atomically smooth mica for a more realistic model of the peptidoglycan layer only accelerates the evolution of the system, with the domains appearing in <3 h. Substrate-biased flip–flop has previously been suggested theoretically^69^ and demonstrated experimentally for phosphocholine membranes, which are more representative of eukaryotic systems.^98^ The resulting spinodal process is reminiscent of demixing observed in ternary lipid systems below the critical points,^110,111^ although with two important differences compared to the present work. First, the absence of physical contacts with a membrane-supporting structure allowed lipid–lipid interactions to dominate the phase behaviour. Second, the presence of cholesterol was key to the formation of solid nanodomains. AFM measurements on ternary phosphocholine-cholesterol SLBs^112^ hint at the unusual dynamics of supported membranes, with the coexistence of three different phases under certain conditions, and the domains forming either by nucleation or spinodal decomposition. At equilibrium, the roughness of the substrate can influence the size of the lipid nanodomains,^113^ further supporting the important role of physical contacts.^56^

Aside from the clear impact of the substrate on the membrane dynamics and molecular organisation, other facts are known to play a role, such as the ionic content of the solution^58^ and the difference of temperature with the transition point. The sealed, humidity-saturated chamber ensures that ionic conditions remain effectively constant throughout each experiment. While this setup does not preclude contributions from individual ions to membrane–substrate coupling, particularly Mg^2+^, which may influence hydration and interactions between the negatively charged membrane and substrate, our primary goal was to replicate the standard ionic composition used for *E. coli* exponential batch culture. Dissecting the specific roles of individual environmental components, given their dual involvement in lipid–substrate interactions and *E. coli* physiology, would constitute a valuable future study. The chamber also limits potential degradation of the sample through *e.g.* lipid oxidation. The impact of temperature variations is clearly illustrated by repeating the experiments at 23 °C and 25 °C. In principle, the AFM tip could also have an impact, both from its mechanical probing of the bilayer and from its photothermal actuation. However, careful temperature control from the base of the sample, with the ability to heat and cool at rates of several degrees per second, mitigates these effects. Generally, the consistency between the experiments, including the significant differences in kinetics between substrates, suggest that tip effects, sample degradation, or unwanted thermal effects do not dominate the fate of the system. Furthermore, tip-induced changes would be localised in a specific area of the sample, something never observed. Generally, the proposed interpretation is motivated not by the kinetics alone, but also by the emergence of a distinct reorganisation regime together with changes in domain height and mechanical properties, and by the strong dependence of this evolution on the supporting environment.

The proposed substrate-biased flip–flop mechanism invoked here to explain the experimental results is not directly observed. Rather, it provides a molecular mechanism consistent with the combined morphological, kinetic and nanomechanical observations and their dependence on the supporting environment. Direct demonstration would require chemical measurements capable of resolving lipid composition separately within the two leaflets at different times. Our attempts to label a single lipid species after the spinodal process has taken place proved unsuccessful (see SI Note S1 for details). Alternatively, techniques such as neutron diffraction,^114^ neutron reflectometry^72^ or nano-Raman^115^ offer some chemical sensitivity, but they are particularly challenging for achieving single leaflet resolution of unmodified SLBs in solution.

The present work remains limited to binary model membranes *in vitro*. While useful to assess the ability of different contact chemistry and geometry to create distinct lipid domains even in the absence of cardiolipin, the prevalence of this effect in natural systems remains to be assessed. Future studies will also need to assess the role of cardiolipin in the same context. However, our results make it possible to identify some of the key thermodynamic and kinetic conditions and characteristics of the process. First, the molecular reorganisation takes place if the membrane has one leaflet in the *S*_o_ phase, which requires it to be close to its *T*_m_ but remain above it in the absence of contacts (here up to ∼8 °C). When below the supported proximal leaflet *T*_m_, the speed of the spinodal process scales with the quenching temperature (Fig. 3), consistent with the idea of a flip–flop-limited evolution. Second, the effect depends on the balance of lipid–lipid and lipid–substrate interactions, as already proposed in previous studies.^56–58,61,65,83,113^ This implies that both the lipid composition of the membrane and the surface chemistry and topography of the contacting surface determine the extent and the kinetics of the molecular reorganisation. Here, different substrates appear to shift the evolution timescales by at least an order of magnitude. Our experiments also highlight the fact that membrane–membrane interactions, which are arguably one of the most common forms of physical contact between biological membranes, can dramatically and rapidly alter the local biophysical properties of the membrane.

The change in the local mechanical properties associated with the spinodal decomposition is considerable and likely has implications for processes such as molecular binding and membrane reshaping. Furthermore, a punctual area of contact, *e.g.* anchoring or support point such as those of FtsZ ring^116^ and MreB actin-like filaments,^50^ can create a local singularity in terms of diffusion or geometry, which in turn may enhance the flip-flopping rate and trigger a rapid local molecular reorganisation.

## Conclusion

This study investigates the kinetics associated with the molecular reorganisation of a model binary system for *E coli*'s inner membrane. Comparing SLBs on different substrates with suspended vesicles evidences a complex evolution of the SLBs over substrate-dependent timescales, resulting in nanoscale lipid domains with different molecular organisation and physical properties. This evolution is not observed for the suspended membranes, pointing to the changes being induced by water-mediated contacts with the different substrates. The existence of such a general mechanism for the spontaneous formation of nanodomains in prokaryotic membranes could have far-reaching consequences for our understanding of their organisation and function, with the present work providing novel insights into their native morphology, including the recently discovered role they play in actively bearing osmotic pressure.^3,46^ The role of ‘contact-induced’ molecular reorganisation in more realistic biological membranes and *in vivo* will need to be assessed in the future, but the characterized impact of the spinodal process on the biophysical properties of the membrane provided in this work suggests it could be highly relevant for understanding the function of prokaryotic membranes.

## Author contributions

TP and KV designed the project. NT performed the experiments and analysed the data with input from TP and KV. All authors commented on the results and wrote the manuscript.

## Conflicts of interest

There are no conflicts to declare.

## Supplementary Material

SM-OLF-D6SM00923A-s001

## Data Availability

The data for this article, including all the data presented in the main manuscript and supplementary information (SI) figures and tables are available at Durham University Research Data Repository at https://researchdata.durham.ac.uk/files/r2hq37vn69j (DOI: https://doi.org/10.15128/r2hq37vn69j). Supplementary information includes supplementary Fig. S1–S9, a note describing the attempt at chemically labeling the membrane and supplementary references. See DOI: https://doi.org/10.1039/d6sm00923a.
