# Supplementary material for: Contact-induced molecular reorganisation in a model Escherichia coli lipid membrane
Source: Soft Matter. 2026 Oct 21. Online ahead of print. doi: 10.1039/d6sm00923a (PMC13742302; doi:10.1039/d6sm00923a)
Supplement: Supplementary file 1 [file SM-OLF-D6SM00923A-s001.pdf]

Supplementary Information (SI) for Soft Matter.
This journal is © The Royal Society of Chemistry

Supplementary Information for

Contact-induced molecular reorganisation in a model *Escherichia coli* lipid membrane

Nicolo Tormena[1], Teuta Pilizota[2,3,*], Kislon Voitchovsky[1,*]

1. Physics Department, Durham University, South Road, Durham DH1 3LE, UK
2. School of Biological Sciences and Centre for Engineering Biology, The University of Edinburgh, Alexander Crum Brown Road, Edinburgh, EH9 3FF, UK
3. Department of Physics, University of Cambridge, JJ Thomson Avenue, Cambridge CB3 0HE

Correspondence: tp579@cam.ac.uk, kislon.voitchovsky@durham.ac.uk

This PDF file includes:

- Supplementary Figures S1 to S9
- Supplementary Note 1: Attempt to chemically label the membrane to track its molecular organization
- Supplementary References

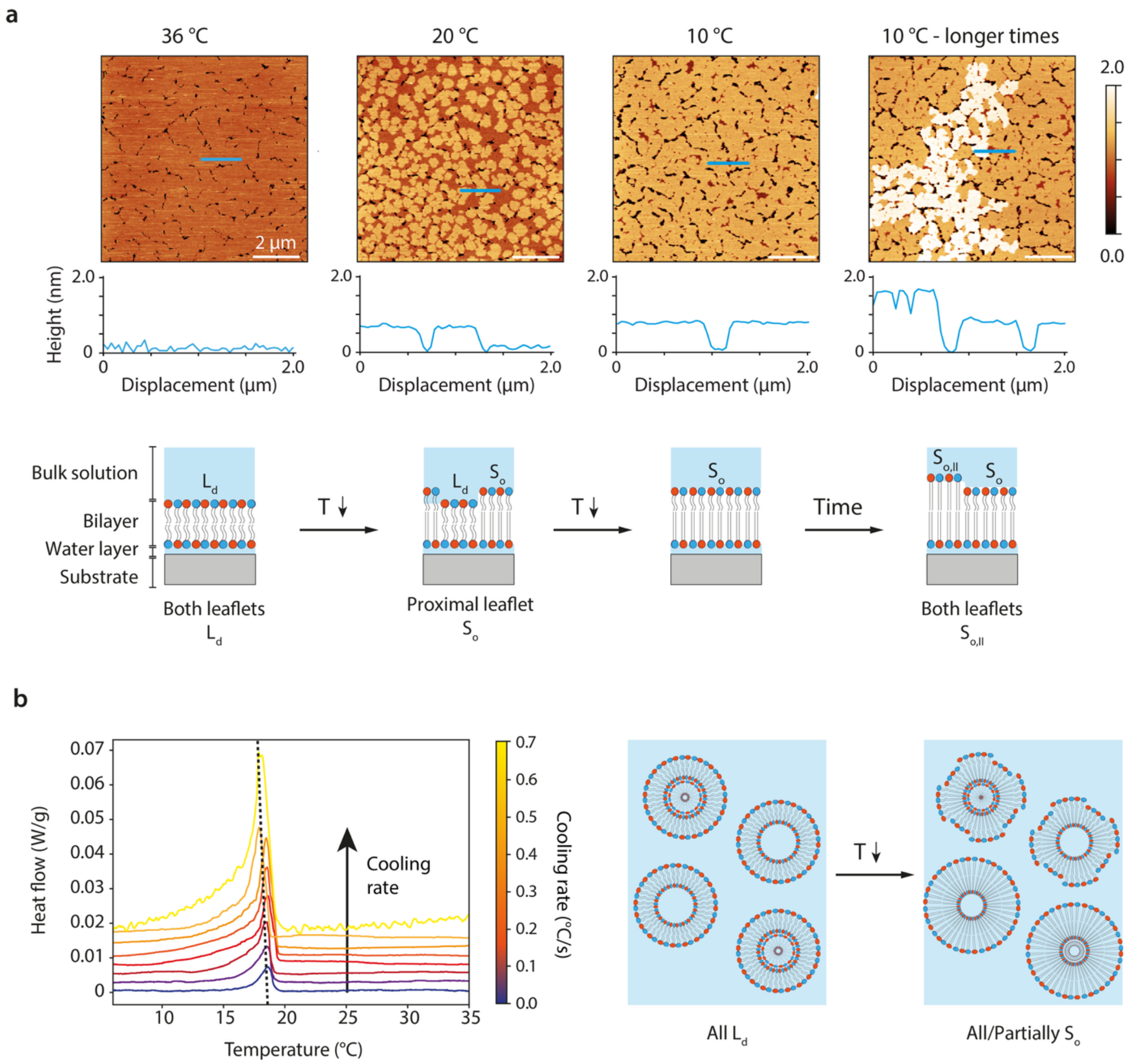

Figure S1: Comparison of the phase transition for supported and suspended bilayers. (a) AFM microscopy imaging of SLBs upon cooling reveals the expected leaflet by leaflet decoupled transition[1–4]. The proximal leaflet transitions at higher temperatures due to stabilizing interactions with the substrate and reduced configurational entropy. When the proximal lipids arrange into the S_o phase, the corresponding membrane domains appear taller (here illustrated at 20°C). The transition is fully complete when both leaflets are in S_o phase, requiring lower temperatures than for the proximal leaflet (here at 10°C). The process is illustrated as a cartoon. In contrast, a single transition is observed for suspended vesicles in solution (b). While DSC thermograms on lipid vesicles at different cooling rates show a small peak shift to lower temperatures as the cooling rate increases, only one peak is visible. The curves have been vertically offset for clarity. A cartoon of the measured LVs is shown, with the sample containing both unilamellar (ULVs) and multilamellar vesicles (MLVs) in solution undergoing the phase transition.

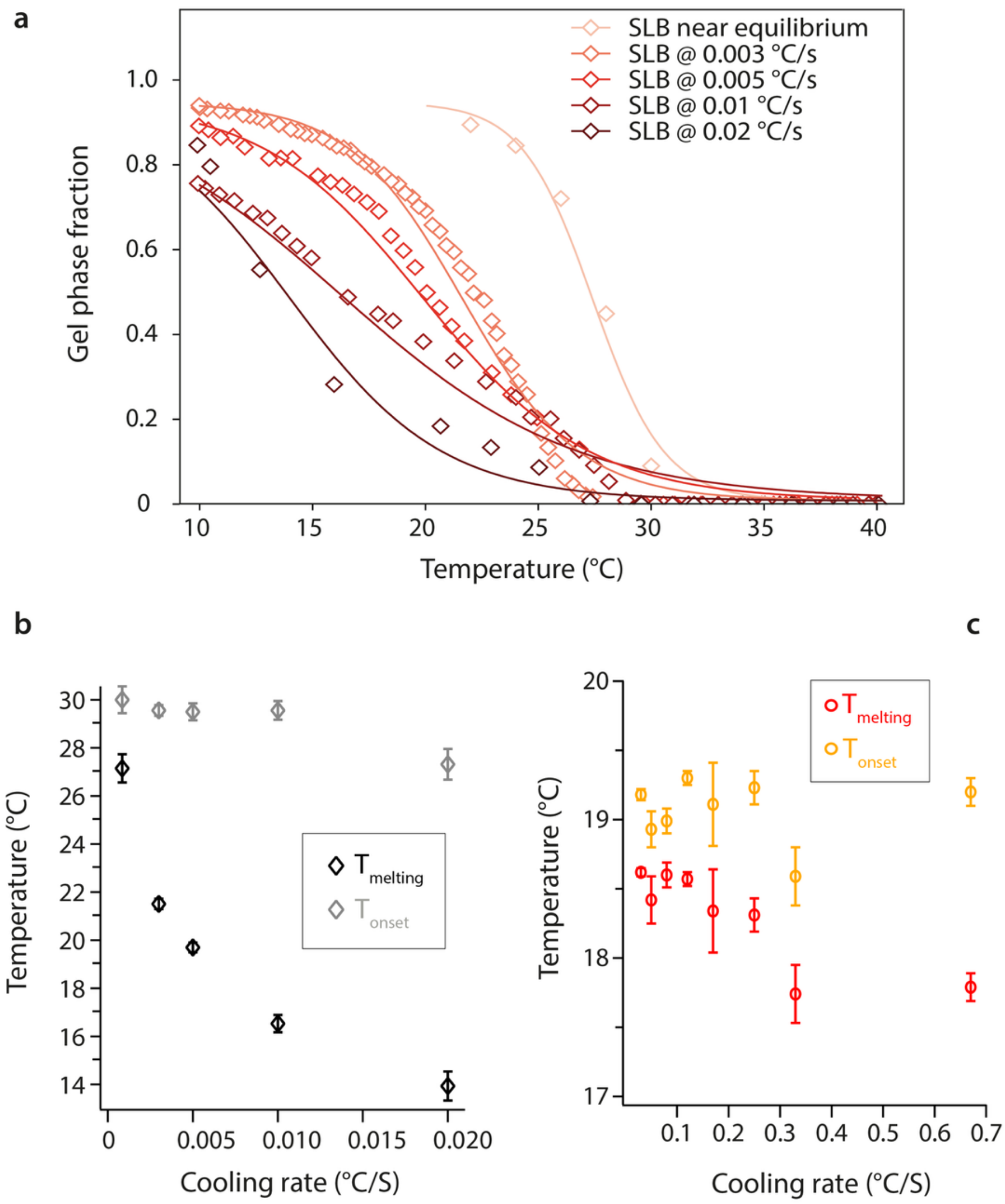

Figure S2: Kinetics of the phase transition for SLBs and suspended membranes. On SLBs (a), the transition kinetics, temperature T_m and enthalpy is obtained by fitting the thermograms to the Van't Hoff equation (1):

$$\ln(k) = \frac{\Delta H_{vH}}{R}\left(\frac{1}{T_m} - \frac{1}{T}\right) \qquad (1)$$

where ΔH_{vH} is the Van't Hoff enthalpy associated with the reaction, and k the equilibrium constant for the S_o to L_d transition (calculated from the gel phase fraction). Experimental data (diamond marks) are displayed with the fitting curves (lines) in each condition. The near equilibrium condition represents a quasi-static cooling experiment where temperature was decreased every 15 min by 2°C. The derived T_m and T_{onset}, which is the temperature at which the transition is initiated, are shown in (b) for each cooling rate. The T_m values derived for the SLBs are highly sensitive to cooling rate, exhibiting a maximal variation of 13 °C.
The equivalent analysis conducted on the suspended vesicles (ULVs and MLVs) is shown in (c). The graphs represent the average with standard deviation for each condition.

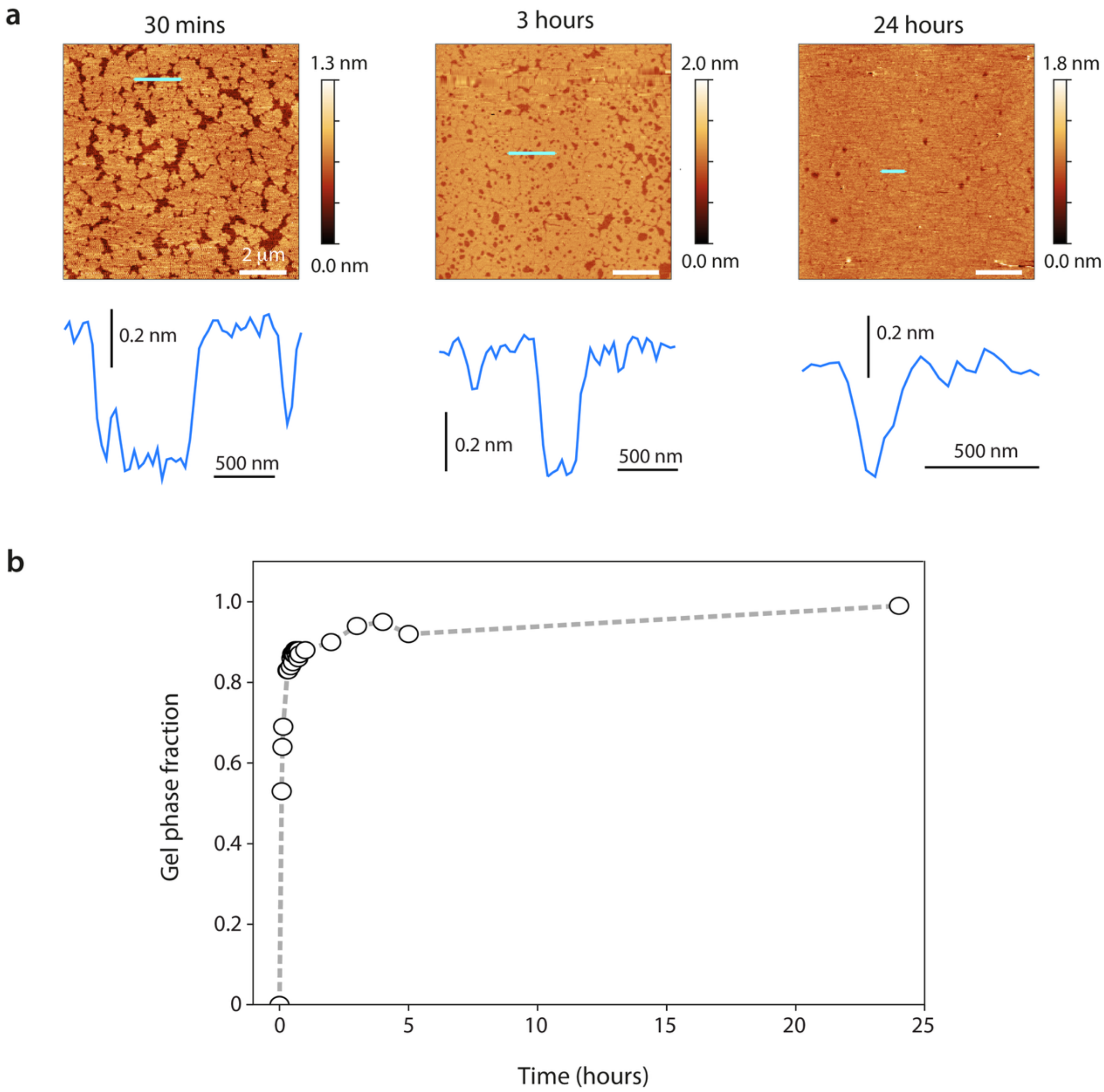

Figure S3: Control experiment on a pure POPE SLB. The SLB undergoes a first order phase transition process within few hours and does not present the same macroscopic morphological features of binary system. (a) Examples of AFM topographical images of POPE SLB at 30°C at different time points after the temperature quench. Each image has been presented with a representative height profile. (b) Gel phase fraction of POPE membrane growth against time after temperature quench. Approximately 98% of the surface is S_o phase 24 h from the start. Both the formation of the SLB and the imaging procedures followed the same protocols described in the *Materials and Methods* section for the binary mixture.

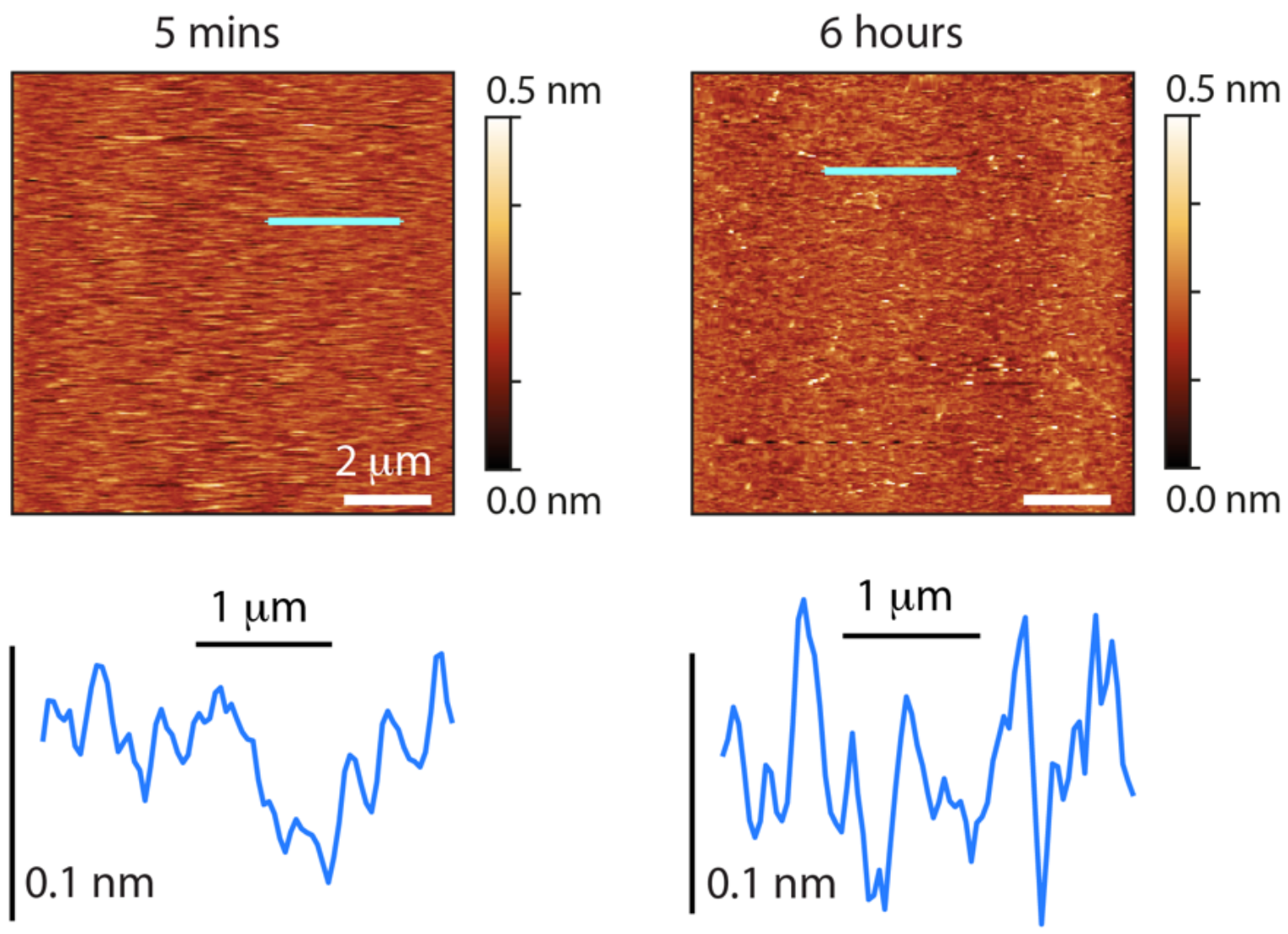

Figure S4: Model *E. Coli* membrane evolution when kept fully in fluid state. AFM images of the binary lipid mixture on mica at 32°C at different times indicates no evolution in structure and topography This is confirmed by the exemplar profiles. The temperature has been selected to be close to the mixture's T_m, but high enough not to trigger the phase transition of the proximal leaflet.

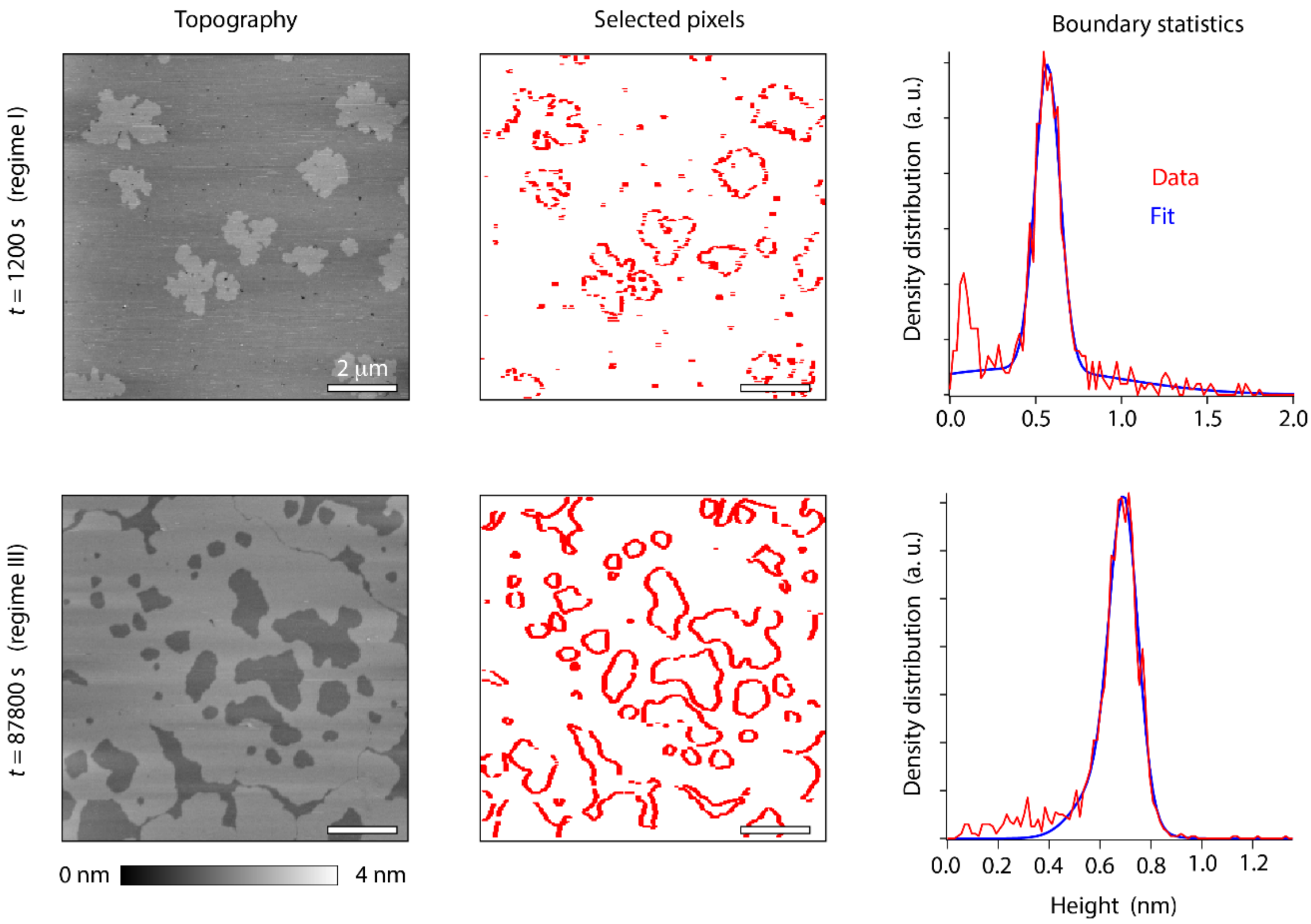

Figures S5: Analysis workflow for domain height differences. From an AFM image corrected for sample tilt (average profile subtraction), a Kirsch edge detection[5] is applied to identify the boundary between the domains of different height. Additional pixels are included each side of the identified edge along the scanning direction (here horizontally) to ensure meaningful statistics (the selected pixels used in the statistics are highlighted in red). For each scan line (horizontal line), the maximum height variation recorded across a given boundary (red) constitutes one measurement. The distribution of all the height variation measurements obtained over a given image provides statistical value for the average height variation across a boundary (Boundary statistics). This approach, which focuses only on the height variation at the boundary as experienced by the scanning AFM tip, ensures minimal impact of the image correction (tilt) over the result. The process is repeated for each image in a semi-automated procedure, programmed here in Igor Pro. The analysis is illustrated here for the early stages of the experiment (top) and late stages (bottom).

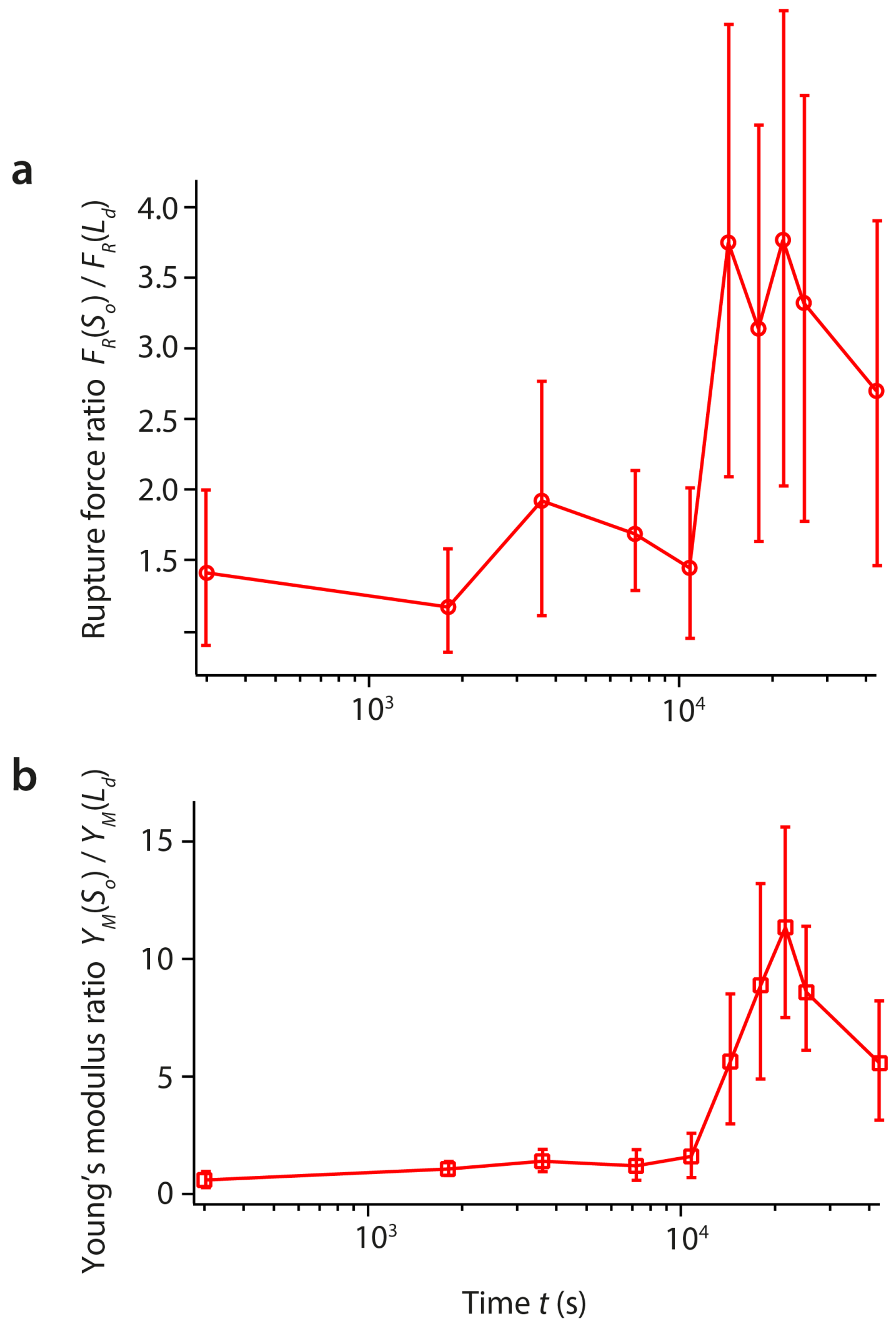

Figure S6: Ratio of the rupture force and Young's modulus measured for the *S_o* and fully *L_d* proximal domains. The absolute values are shown in Fig. 2e-f with the ratio presented here evidencing the differential evolution of the two domains types. Considering the ratio has the advantages to mitigate for a possible evolution in the geometry of the AFM tip over the course of the experiment. This is because the same tip is used for all the nanomechanical measurements, comprising both types of domains at any given time. The error bars are calculated from Fig. 2e-f.

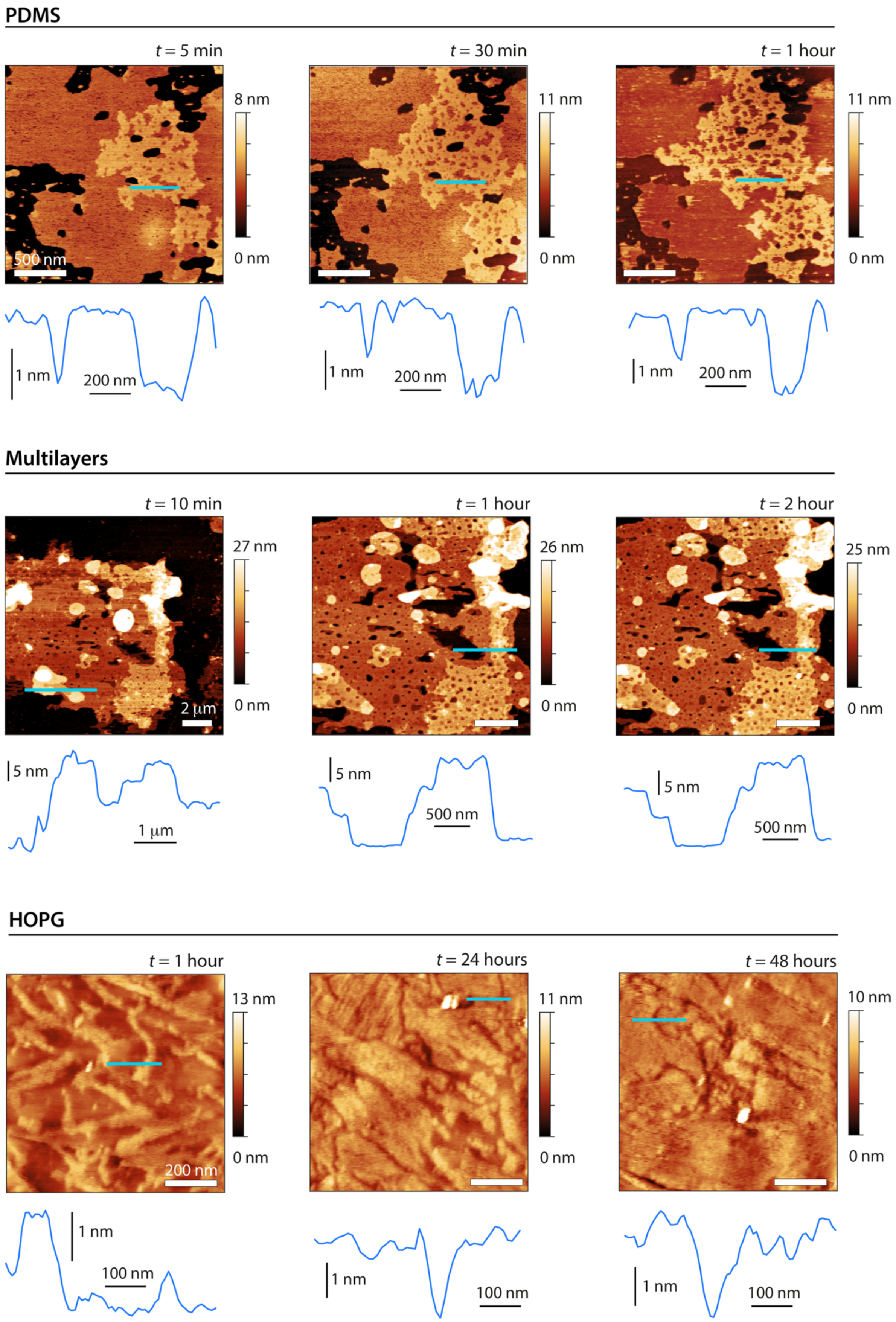

Figures S7: Full field of view AFM images and profiles of the samples shown in Fig. 3. The images are of the samples' topography, and representative profiles are shown immediately under each image.

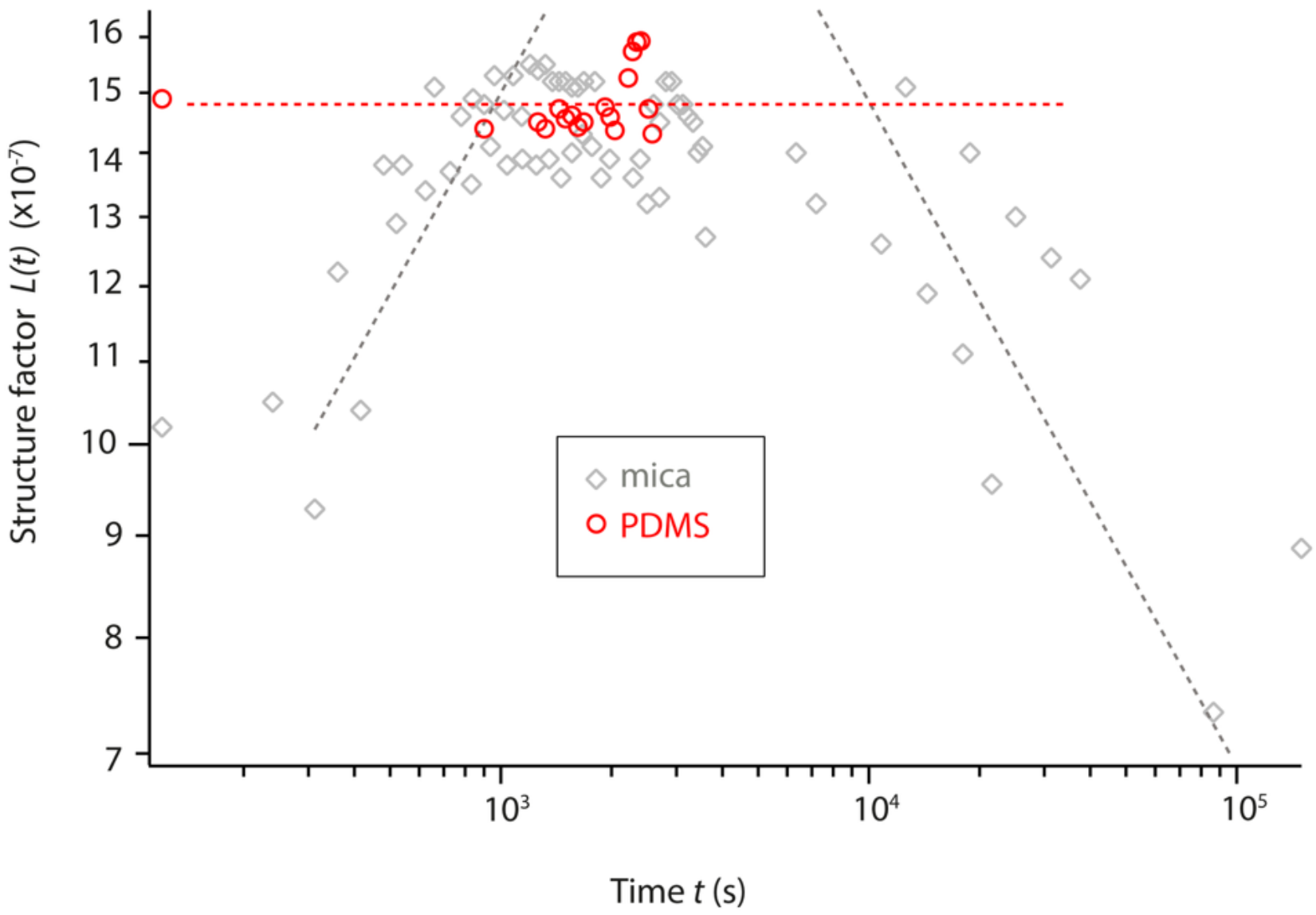

Figure S8: Log-log of the characteristic length scale $L(t)$ vs t for SLBs formed on mica and PDMS. In both cases, the lipid domains analysis has been done following the same principles, with indicative dashed lines drawn to guide the eye. The PDMS has been rescaled by a factor 2.8 to aid visualisation on a same vertical axis as the data collected on mica. Given the log-log representation, this rescaling simply induces a vertical offset of the data. On PDMS the system appears to have completed its evolution from the onset of AFM imaging ($\alpha \sim 0$). The domains evolution for the bilayer on bilayer structures was even faster and could not be reliably captured by AFM.

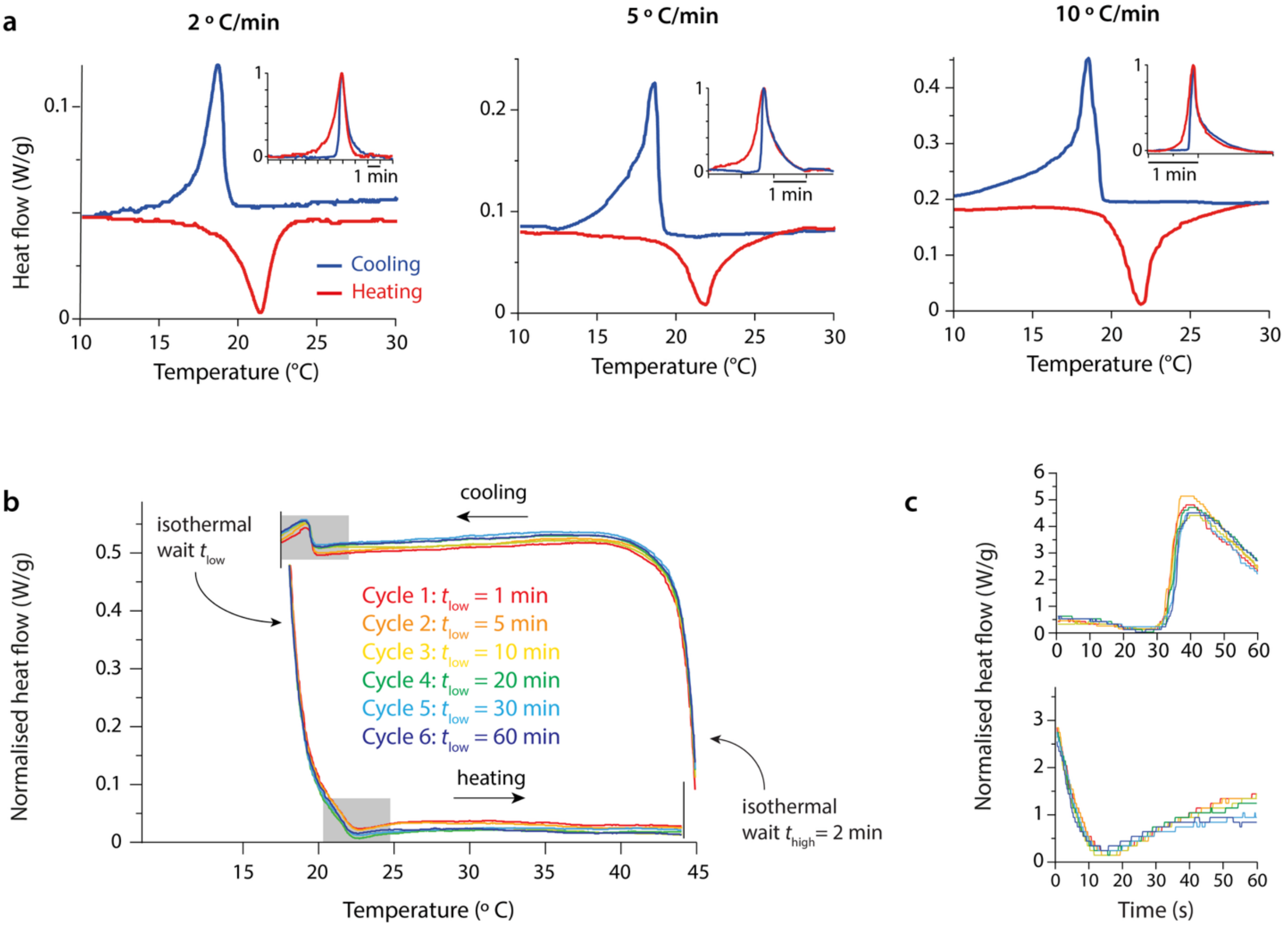

Figure S9: Comparison of cooling and heating phase transition curves obtained on LVs and measured by DSC. (a) Thermograms are recorded during cooling and heating at 2 °C/min, 5 °C/min and 10 °C/min. Insets display the normalized heating and cooling curves overlaid to highlight differences or similarities in their shapes. To test a possible slower evolution of the membrane, DSC cycles were conducted with different waiting times at ~2 °C below T_m in (b). Normalized heat flow is obtained from a LV sample first cooled at 5 °C/min (slow enough to allow for membrane equilibration) and kept isothermally for a duration t_{low} at ~2 °C below T_m. The sample is then re-heated to control the impact of the isothermal wait on the phase transition properties of the LVs. Six consecutive cycles with different waiting times were conducted, each indicated with a different colour. The cooling and heating transitions are highlighted in grey shaded rectangles. (c) Magnification of the cooling and heating transition peaks show no clear differences between the different cycles, within error, but quantization of the signal (heat flux) indicates that the measurements are at the limit of the calorimeter's sensitivity.

Supplementary Note 1: Attempt to chemically label the membrane in order to track its molecular reorganisation

Testing the driving force behind the morphological evolution of our SLB systems directly is not trivial, as this would require tracking the movement of single molecules within the bilayer. To observe this hypothesized compositional variation, we attempted to singularly track one of the two lipid species over the different phase transition stages. The first approach involved incubating the membrane, at different time intervals, using cinnamycin, which is an antibacterial peptide able to recognize PE headgroup[6,7]. Although cinnamycin adsorption of the bilayer was observable with AFM, the marker did not spread homogenously across the surface even at high temperature where the mixture was perfectly mixed. Cinnamycin tended to accumulate mainly along bilayer defects, likely due to interactions between it and the underlying surface. As an alternative, we employed cesium ions (positively charged), which we hypothesized would preferentially interact with PG headgroups (negatively charged) due to the favourable electrostatic interactions. Given cesium's large radius, we expected to observe some topographical variations while imaging the sample with AFM. To do that, we worked with a ratio between the set-point amplitude and the free oscillation amplitude greater than 0.6, generally used to perform gentle and high-resolution imaging on AFM[8]. Unfortunately, under these conditions, the height and phase images showed no resolvable differences or contrast that could have been attributed to the cesium interaction. he absence of such features is likely due to the weak electrostatic interactions between cesium ions and the membrane, which allow the ions to be displaced by vertical and lateral perturbations from the AFM tip. Changing the solution's pH would have increased the strength of these interactions but inevitably modifying the phase transition properties. Another possible way forward could, in principle, use fluorescently labelled lipids. However, this would significantly change the system's properties, in particular the flip-flop rate in the SLBs and the lipid-lipid interactions. We therefore did not attempt it.

Supplementary References

1 A. Charrier and F. Thibaudau, *Biophys. J.*, 2005, **89**, 1094–1101.

2 H. M. Seeger, G. Marino, A. Alessandrini and P. Facci, *Biophys. J.*, 2009, **97**, 1067–1076.

3 O. Enders, A. Ngezahayo, M. Wiechmann, F. Leisten and H.-A. Kolb, *Biophys. J.*, 2004, **87**, 2522–2531.

4 D. Keller, N. B. Larsen, I. M. Møller and O. G. Mouritsen, *Phys. Rev. Lett.*, 2005, **94**, 025701.

5 W. K. Pratt, *Digital Image Processing*, John Wiley & Sons, Inc., 4th ed., 2006.

6 G. Machaidze and J. Seelig, *Biochemistry*, 2003, **42**, 12570–12576.

7 M. Vestergaard, N. A. Berglund, P.-C. Hsu, C. Song, H. Koldsø, B. Schiøtt and M. S. P. Sansom, *ACS Omega*, 2019, **4**, 18889–18899.

8 E. J. Miller, W. Trewby, A. F. Payam, L. Piantanida, C. Cafolla and K. Voïtchovsky, *JoVE*, 2016, e54924.
